# Identification and Expression Profiling of WRKY Family Genes in Sugarcane in Response to Bacterial Pathogen Infection and Nitrogen Implantation Dosage

**DOI:** 10.3389/fpls.2022.917953

**Published:** 2022-06-09

**Authors:** Talha Javed, Jing-Ru Zhou, Juan Li, Zhong-Ting Hu, Qin-Nan Wang, San-Ji Gao

**Affiliations:** ^1^National Engineering Research Center for Sugarcane, Fujian Agriculture and Forestry University, Fuzhou, China; ^2^Institute of Nanfan & Seed Industry, Guangdong Academy of Sciences, Guangzhou, China

**Keywords:** *Saccharum* spp., WRKY transcription factors, gene regulation, *Xanthomonas albilineans*, nitrogen dosage

## Abstract

WRKY transcription factors (TFs) are essential players in different signaling cascades and regulatory networks involved in defense responses to various stressors. This study systematically analyzed and characterized WRKY family genes in the *Saccharum* spp. hybrid R570 and their expression in two sugarcane cultivars LCP85-384 (resistant to leaf scald) and ROC20 (susceptible to leaf scald) in response to bacterial pathogen infection and nitrogen implantation dosage. A total of 53 *ShWRKY* genes with 66 alleles were systematically identified in R570 based on the query sequence *SsWRKY* in *S. spontaneum* AP85-441. All *ShRWKY* alleles were further classified into four groups with 11 (16.7%) genes in group I, 36 (54.5%) genes in group II, 18 (27.3%) genes in group III, and 1 (1.5%) gene in group IV. Among them, 4 and 11 *ShWRKY* gene pairs displayed tandem and segmental duplication events, respectively. The *ShWRKY* genes exhibited conserved DNA-binding domains, which were accompanied by variations in introns, exons, and motifs. RT-qPCR analysis of two sugarcane cultivars triggered by *Xanthomonas albilineans* (*Xa*) revealed that four genes, *ShWRKY13-2/39-1/49-3/125-3*, exhibited significant upregulation in leaf scald-resistant LCP85-384. These WRKY genes were downregulated or unchanged in ROC20 at 24–72 h post-inoculation, suggesting that they play an important role in defense responses to *Xa* infection. Most of the 12 tested *ShWRKY*s, *ShWRKY22-1/49-3/52-1* in particular, functioned as negative regulators in the two cultivars in response to a range of nitrogen (N) implantation doses. A total of 11 ShWRKY proteins were predicted to interact with each other. ShWRKY43 and ShWRKY49-3 are predicted to play core roles in the interaction network, as indicated by their interaction with six other ShWRKY proteins. Our results provide important candidate gene resources for the genetic improvement of sugarcane and lay the foundation for further functional characterization of *ShWRKY* genes in response to coupling effects of *Xa* infection and different N levels.

## Introduction

Sugarcane (*Saccharum* spp.) is an important industrial C_4_ crop that accounts for 80% of sugar and 40% of bioethanol production globally (Aono et al., 2021). Various biotic stresses are major factors that can impede the whole growth and development period of sugarcane, resulting in 10–15% yield losses worldwide (Barnabas et al., 2015). Leaf scald caused by *Xanthomonas albilineans* (*Xa*) is a main bacterial disease in sugarcane and produces severe abnormalities, such as stunted growth, chlorosis, necrosis in leaves, and even death of the entire plant (Hong et al., 2021). On the other hand, stress induced by nitrogen (N) imbalance is a curial abiotic factor for plant growth and production of crops, including sugarcane (Yang et al., 2019; Vidal et al., 2020). Insufficient or ill-timed application of N fertilizer leads to poor growth, whereas excessive application of synthetic N fertilizer, especially during the later growth stage, delays the phase transition from vegetative to reproductive growth and ultimately decreases the sugar content (Yang et al., 2019). Excessive application of N fertilizer also increases the frequency of disease and pest infestation of crops and results in acidic soil, eutrophic water, and non-point pollution as well as increased production costs (Shrivastava and Srivastava, 2012; Yang et al., 2019).

Transcription factors (TFs) activate different signal transduction cascades and modulate the transcriptional efficiency of targeted genes to play a crucial role in adapting crops to different environmental constraints (Baillo et al., 2019; Javed et al., 2020; Hu and Sperotto, 2021). WRKY TFs that are important plant-specific regulatory genes are characterized by one or two conserved WRKY domains (WDs) in the N terminus that are usually followed by a zinc-finger motif at the C terminus (Wani et al., 2021). These two motifs play a crucial role in the WRKY DNA-binding domain (DBD) (Chen et al., 2017; Wani et al., 2021). WRKY TFs bind to (T)(T)TGAC(C/T) (i.e., W-box) *cis*-acting elements in the promoter of target genes and subsequently modulate gene expression (Chen et al., 2017). Meanwhile, the zinc-finger-like motif plays a critical role in the evolution of plants (Eulgem et al., 2000; Chen et al., 2019). Based on the number of WDs and the type of zinc-finger motifs, WRKY proteins can be divided into four major groups: Group I comprises two WDs and a single C_2_H_2_ zinc finger; groups II and III have one WD with a C_2_H_2_ zinc finger and C_2_HC zinc finger, respectively (Chen et al., 2019). Group IV having an incomplete/partial WD (only the WRKYGQK motif was observed) and lacking a zinc-finger motif was proposed for *S. spontaneum* AP85-441, suggesting that members of this group may have lost their function as WRKY TFs (Li et al., 2020). The WRKY groups have several subgroups. For example, the group I can be classified into two subgroups Ia and Ib, which have two WDs with C_2_H_2_ zinc fingers at the N and C terminus, respectively (Chen et al., 2019; Li et al., 2020). Group II is divided into five subgroups (IIa-IIe) according to additional conserved motifs located adjacent to the WD, whereas group III is separated into subgroups IIIa and IIIb based on the zinc-finger motif structure (Eulgem et al., 2000; Chen et al., 2019).

An increasing number of studies revealed that WRKY TFs play a pivotal role in the response of plants to pathogen infection and stressful nutrient conditions (Phukan et al., 2016; Wani et al., 2021). For instance, comparative transcriptome analysis of resistant and susceptible cultivars evidenced the upregulated expression of *WRKY33* alleles in sugarcane with *Xa* infection (Ntambo et al., 2019). Tomato plants overexpressing *SlWRKY8* showed resistance against *Pseudomonas syringae* pv. tomato DC3000 (Pst DC3000) by increasing transcription of two pathogen-related genes *SlPR7* and *SlPR1a1* (Gao et al., 2020). In rice, the gene *OsWRKY93* has dual functions in both leaf senescence and in response to *Magnaporthe oryzae* infection (Li et al., 2021). Transgenic grapevine plants overexpressing *VqWRKY31* have enhanced resistance to powdery mildew caused by the fungal pathogen *Erysiphe necator* by promoting salicylic acid signaling and specific metabolite synthesis (Yin et al., 2022). A recent study demonstrated that >64% of *AktWRKY* genes from *Akebia trifoliata* were differentially expressed during *Colletotrichum acutatum* infection in two varieties I02 (susceptible) and H05 (resistant) (Wen et al., 2022). In addition, WRKY TFs contribute to processes associated with nutrient deprivation. For instance, four WRKY genes (*AtWRKY6/42/45/75*) from *Arabidopsis* and two genes (*OsWRKY74/80*) from *Oryza sativa* are involved in plant nutrient utilization, including phosphorus, boron, and iron (Chen et al., 2017). N treatment was shown to enhance the production of sterols and withaferin A through transcriptional activation of the jasmonate pathway and WRKY TFs in *Withania somnifera* (Pal et al., 2017). An integrated analysis of the rice transcriptome and metabolome revealed the upregulation of six *OsWRKYs* in response to low N supply (Xin et al., 2019). The protective effects of silicon against low phosphorus stress in tomato plants might affect the expression of WRKY TFs (Zhang et al., 2021). Notably, recent observations support the involvement of WRKY-mediated crosstalk between abiotic and biotic stress responses (Wani et al., 2021). A codependent behavior was observed between septoria leaf blotch and low N availability that involved altered WRKY TF expression (Poll et al., 2020).

Recently, genome-wide identification of WRKY family genes was performed in the autopolyploid *S. spontaneum* AP85-441, and temporal and spatial patterns of these *SsWRKYs* were examined in different tissues at developmental stages based on RNA-seq data (Li et al., 2020). However, little is known about WRKY family genes in sugarcane in response to bacterial pathogen infection and N implantation dosage. This study identified and characterized WRKY family genes in the *Saccharum* spp. hybrid R570 and also included expression profiling of two cultivars LCP85-384 (leaf scald-resistant) and ROC20 (leaf scald-susceptible) with *Xa* infection and different N implantation dosages. Our results expand our understanding of how *ShWRKY* genes play dual functions between N and *Xa*-stress responses in sugarcane.

## Materials and Methods

### Crop Husbandry

The two cultivars LCP85-384 (resistant to leaf scald) and ROC20 (susceptible to leaf scald) used for this study were procured from the National Engineering Research Center for Sugarcane, Fujian Agriculture and Forestry University, Fuzhou, China (26.0849° N, 119.2397° E). Sugarcane stems were cut as single-budded setts that were immersed under flowing water for 24 h at room temperature and then treated with hot water (50°C) for 2 h. The setts were dried at room temperature for 2 h before sowing. Peat soil (PINDSTRUP, Denmark) containing NH_4_NO_3_ (33 g/m^3^), pH (5.5), K_2_O (158 g/m^3^), and P_2_O_5_ (91 g/m^3^) was used for the experiments. Sugarcane seedlings were grown for 28 days (3–5 leaf stage) in a climatic chamber set at 28°C with a 16/8 h light/dark cycle and 60% relative humidity.

### *Xanthomonas albilineans* Inoculation, Nitrogen Application, and Leaf Sampling

The bacterial strain Xa-FJ1 (Zhang et al., 2020) was suspended in 1 ml XAS solution at 28°C for 48 h with shaking at 200 rpm. Suspended cells (1 μl) were added to freshly prepared XAS solution (40 ml) and cultured for 10 h at 28°C. For plant inoculation, the bacterial cultures were diluted to 10^8^ CFU/ml and inoculated using a leaf-cutting method (Ntambo et al., 2019). Urea (46% N) as an N source was procured from BIOFOUNT, China. For the three N treatments, 2.50 g (N1), 5.00 g (N2), or 7.50 g (N3) was added to the pots (Yang et al., 2019). Leaf samples for both *Xa* infection and N dosage were collected 0, 24, 48, and 72 h later. The harvested leaves were quickly placed in liquid nitrogen and stored at −80°C for subsequent analysis of RNA extraction, followed by RT-qPCR.

### Identification of WRKY Genes in *Saccharum* spp. Hybrid R570

*SsWRKY* genes from *S. spontaneum* (Li et al., 2020) were used as query sequences to search for WRKY family genes in the genome database of *Saccharum* spp. hybrid R570^1^. The candidate sequences corresponding to query sequences that had ≥80% similarity and zero *e*-value were selected for domain checking using NCBI BLASTP^2^, Pfam^3^, and Simple Modular Architecture Research Tool (SMART) domain analysis software^4^ after manually removing redundant sequences. Sequences lacking the WRKYGQK domain were excluded manually before analysis. The nomenclature of WRKY family genes from the R570 genome corresponded to Li et al. (2020).

### Physio-Chemical Properties

Physio-chemical attributes, such as protein molecular weight (MW), number of amino acids (aa), theoretical isoelectric point (pl), instability index (II), aliphatic index (AI), and grand average of hydropathicity (GRAVY), were computed using the ExPASy Proteomics Server^5^. To predict the subcellular localization of genes, the CELLO2GO web server^6^ was used.

### Multiple Sequence Alignment and Phylogenetic Analysis

Multiple sequence alignment of WRKY proteins was carried out using the CLUSTALW algorithm with default parameters in MEGA 7.0 software (Kumar et al., 2018). The phylogeny of aligned sequences was constructed using MEGA 7.0 software with the neighbor-joining method and bootstraps of 1,000 replicates.

### Protein-Protein Interaction, Gene Structure, and *Cis*-Regulatory Elements Analysis

Protein-protein interaction networking of WRKY family genes according to their orthologs in *A. thaliana* was predicted using the STRING database^7^. To determine the localization and lengths of introns, exons, and untranslated regions (UTRs), the Gene Structure Display Server^8^ was used. The MEME tool^9^ was used to determine conserved motifs, followed by visualization with TBtools (Toolbox for biologists) v0.6655 (Chen et al., 2020). The number and distribution of motif sites were set at 10 motifs and zero and one occurrence per sequence, respectively. The PlantCARE database was used to analyze *cis*-regulatory elements for each gene analyzed beginning from the start codon to 1.5 kb upstream^10^.

### Chromosomal Distribution, Gene Duplication, Collinearity, and Ka/ks Analysis

To determine the chromosomal distribution of WRKY family genes, gff3-files extracted from the *Sorghum bicolor* genome^11^ were used to map the genes to respective chromosomes with TBtools v0.6655. Later, the renamed file from the R570 genome was used according to the nomenclature. TBtools v0.6655 was also used to determine gene duplication events and to conduct collinearity analysis among WRKY family genes from *S. spontaneum* AP85-441 and *Saccharum* spp. hybrid R570. The easy_KaKs calculation program was used to determine non-synonymous (Ka) and synonymous (Ks) substitution ratios^12^.

### Expression Profiling Using RNA-Seq Data

A previously published RNA-seq dataset (accession number PRJNA549590) was used to determine WRKY gene expression in two sugarcane cultivars (Ntambo et al., 2019). The fragments per kilobase of transcript per million fragments mapped (FPKM) value for each gene was calculated and then transformed to log_2_ (Fold Change) values for the generation of a heatmap with TBtools v0.6655.

### WRKY Gene Expression Analysis by RT-qPCR

Megazol reagent (Invitrogen, United States) was used to extract RNA from leaf samples according to the manufacturer’s instructions. After checking RNA quality and concentration, the PrimeScript™ RT Reagent Kit was used for reverse transcription following the manufacturer’s protocol. The synthesized cDNA was diluted to 100 ng/μl for qPCR. Gene-specific primer pairs were designed using the GeneScript^®^ tool^13^ (Supplementary Table 1). RT-qPCR was carried out using ChamQ Universal SYBR qPCR master mix (Vazyme, China) on a QuantStudio^®^ Real-Time PCR system (Applied Biosystems, United States). The reaction mixture contained 10 μl 2× ChamQ master mix, 0.4 μl forward primer, 0.4 μl reverse primer, 1 μl cDNA template, and ddH_2_O to reach a 20 μl reaction volume. The following conditions were used for RT-qPCR: denaturation at 95°C for 30 s, followed by 40 cycles at 95°C for 10 s, and 60°C for 30 s. Glyceraldehyde 3-phosphate dehydrogenase (GAPDH) was used as a reference gene. Gene expression was determined by the quantification method (2^–ΔΔCt^). Three biological replicates and three technical replicates were carried out for each sample.

### Statistical Analysis

The means of different time points were compared using the least significance difference (LSD) test at a 5% probability level (*p* ≤ 0.05) with a statistical software package Statistix 8.1^14^.

## Results

### Identification, Phylogeny, and Physio-Chemical Properties of WRKY Family Genes in *Saccharum* spp. Hybrid R570

**FIGURE 1:**
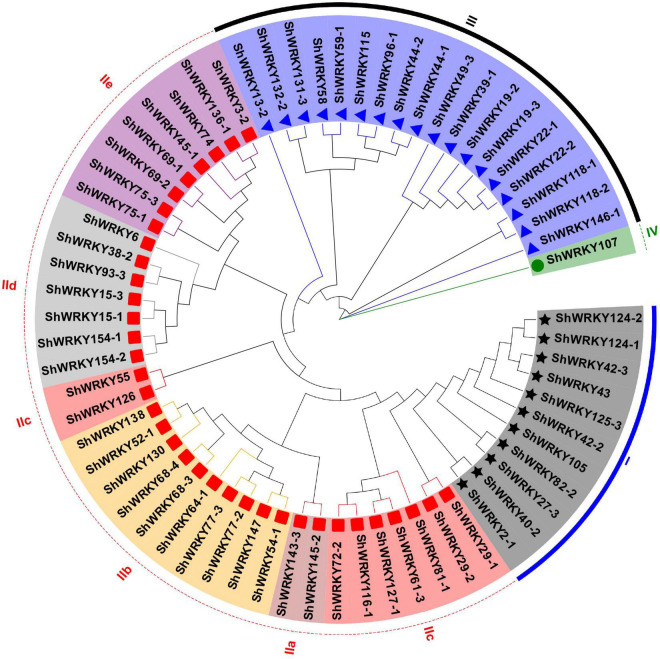
Phylogenetic tree of *ShWRKYs*. The unrooted NJ tree was constructed based on the WRKY domains from *Saccharum* spp. hybrid R570 using MEGA7.0 with bootstraps of 1,000 replicates. Group and subgroup names are on the outer ring and are depicted in different colors. Black stars, red boxes, blue triangles, and green circles represent different *ShWRKY* groups I, II, III, and IV, respectively.

WRKY proteins from *S. spontaneum* AP85-441 (SsWRKYs) were used as query sequences against the R570 protein database to identify WRKY family genes. After excluding redundant sequences and domain confirmation, 53 WRKY genes (*ShWRKYs*) with 66 alleles were finalized in the R570 genome (Supplementary Table 2). Of these, 13 (24.5%) ShWRKYs had one allelic gene. Based on the WD and zinc-finger type, 11 *ShWRKYs* belonged to group I with two WDs, whereas 36 *ShWRKYs* with one WD and one C_2_H_2_ zinc finger belonged to group II. In addition, 18 *ShWRKYs* with one WD and C_2_HC zinc finger belonged to group III. Interestingly, one *ShWRKY* gene (*ShWRKY107*) with a partial WD was assigned to group IV (Figure 1 and Supplementary Table 2). The group II *ShWRKYs* were further divided into five subgroups (IIa-IIe): Subgroup IIa was the smallest group with only two *ShWRKYs* that had a CX_5_CPVKKKVQ motif; whereas IIb and IIc were larger with *ShWRKYs* (9–10 alleles) having a CX_5_CPVRKQVQ and CX_4_C motif, respectively; Subgroup IId had seven *ShWRKYs* with a CX_5_CPARKHVER motif; Subgroup IIe comprised eight *ShWRKYs* with a CX_5_C(P/A/M)ARK(Q/L)VER motif (Supplementary Figure 1). Notably, the group I included some *SsWRKYs* from group III that were identified in the AP85-441 genome (Supplementary Figure 2).

Detailed information about the physio-chemical properties [e.g., length of amino acids, molecular weight, isoelectric point, instability index, aliphatic index, and grand average of hydropathicity, (GRAVY)] of the *ShWRKY* genes are presented in Supplementary Table 2. The ShWRKY proteins had between 249 and 843 amino acids and molecular weights that ranged from 27,131.85 to 95,533.93 kDa, isoelectric points between 4.68 and 10.18, instability indexes ranging from 37.76 to 85.57, and aliphatic indexes between 39.69 and 82.0. Interestingly, the GRAVY values for all WRKY proteins were negative, suggesting the hydrophilic nature of ShWRKYs. The prediction of subcellular localization suggested the presence of *ShWRKYs* in the nucleus except for *ShWRKY69-1*, which was located in the chloroplast (Supplementary Table 2).

### Gene Structure and *Cis*-Regulatory Elements Analysis

**FIGURE 2:**
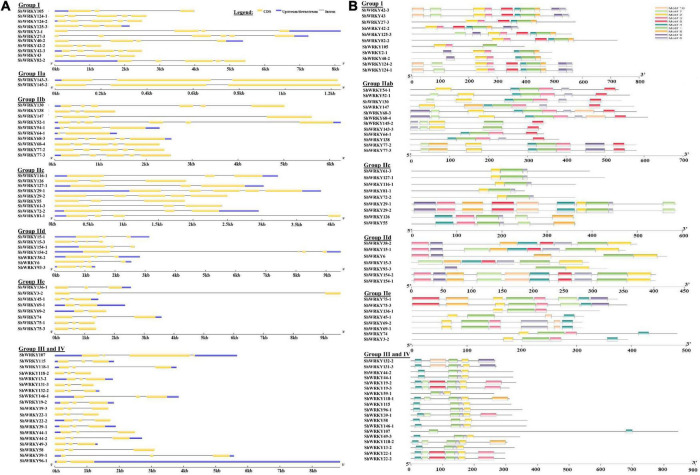
**(A)** Locations and lengths of *ShWRKY* exons and introns which are shown as filled yellow bars and thin gray single lines, respectively. UTRs are represented by dark blue bars at the ends of the lines. Gene structures were drawn using the GSDS online database. **(B)** Conserved motif analysis of *ShWRKYs*. All motifs were identified using the MEME online database and visualized with Tbtools software. A total of ten predicted motifs are represented by different colored boxes and motif sizes can be estimated using the scale at the bottom.

**FIGURE 3:**
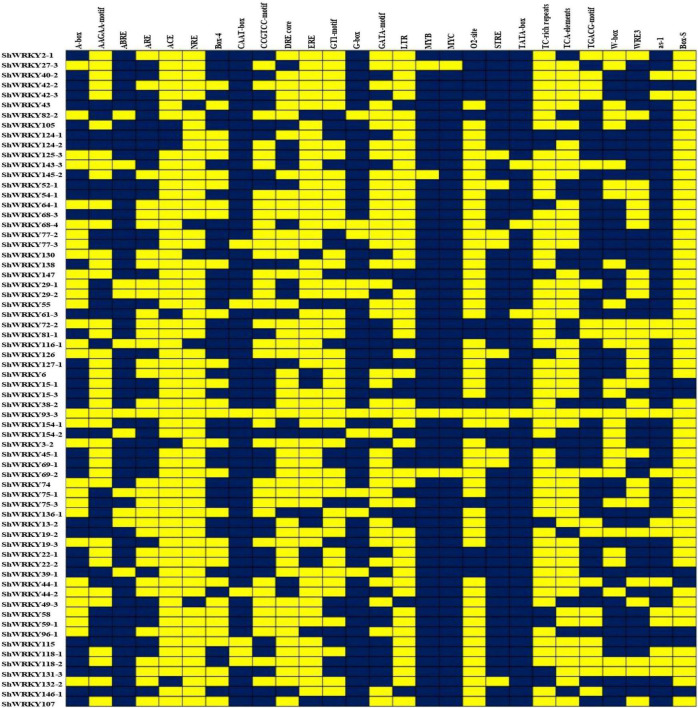
Heatmap of *cis*-regulatory elements in the *ShWRKY* genes. Blue: Present, Yellow: Absent.

Among the 53 *ShWRKYs* (66 alleles), gene structure analysis suggested that the number of introns ranged from 1 (*ShWRKY64-1/69-1/96-1*) to 5 (*ShWRKY27-3/52-1/130*), whereas the number of exons ranged from 2 (*ShWRKY64-1/69-1/96-1*) to 6 (*ShWRKY27-3/52-1/130*). The longest intron structure was observed in *ShWRKY3-2*, followed by *ShWRKY154-2* and *ShWRKY147*. Similar intron (2) and exon (3) distribution patterns were observed in each gene of group III except for *ShWRKY96-1*, which had 1 intron and 2 exons (Figure 2A). Converse motif numbers ranged from 2 (*ShWRKY107*) to 10 (*ShWRKY29-1/42-3/43/124-1/154-1*). Motif 1 was present in all *ShWRKYs* and > 90% had motif 2, which was absent in five genes, *ShWRKY55/64-1/107/126/138* (Figure 2B). A total of 27 *cis*-regulatory elements related to metabolism, seed, endosperm, meristem, stress, light, and hormone responsiveness were predicted to exist in the region 1.5 kb upstream of *ShWRKYs* (Supplementary Figure 3). Importantly, *ShWRKYs* had the highest number of stress-responsive *cis*-elements ranging from 3 (*ShWRKY45-1/145-2*) to 13 (*ShWRKY29-1/147/127-1*) (Supplementary Figure 3), while MYC, MYB, TATA-box, and STRE *cis*-regulatory elements were found in more than 96, 94, 94, and 83% of *ShWRKYs*, respectively (Figure 3).

### Chromosomal Distribution, Gene Duplication, Collinearity, MicroRNA Targeting Site Prediction, and Ka/ks Analysis

**FIGURE 4:**
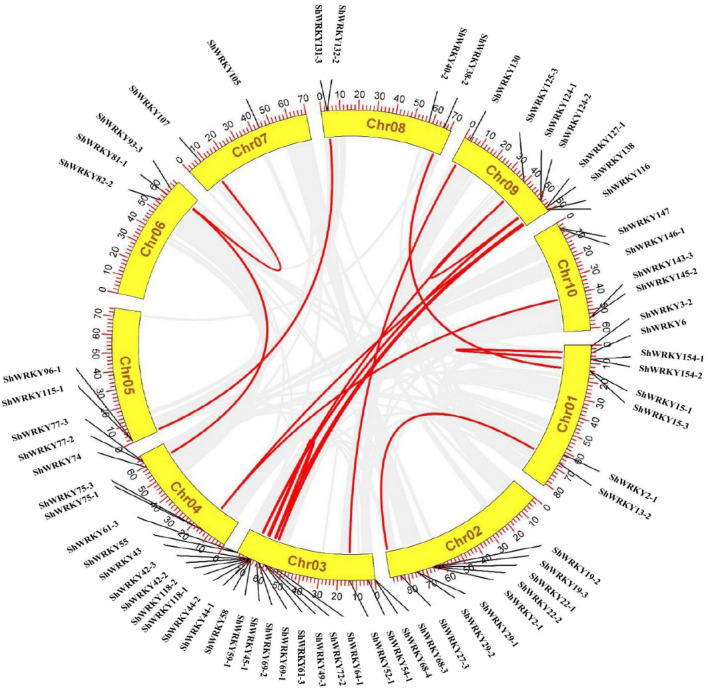
Chromosomal distribution of *ShWRKY* genes in *Saccharum* spp. hybrid R570. Ribbon links (Red lines) indicate segmental duplication events between genes. Chromosome numbers are indicated inside the yellow segments. Chr01 to Chr10 are currently based on *Sorghum bicolor* genome scaffolds (https://phytozome-next.jgi.doe.gov/info/SbicolorSC187_v1_1). The *Saccharum* spp. R570 gff3 file contains these 10 chromosomes and all BAC clones that could not be placed. The gene names on each chromosome are indicated in the outer circle.

Chromosomal distribution revealed that all *ShWRKYs* were mapped on 10 chromosomes of the R570 genome (Supplementary Figure 4). Chromosome 3 had the most *ShWRKY*s (17), and individual chromosomes 1 and 2 each had 7. Chromosomes 5 and 7 had the fewest *ShWRKYs* (*ShWRKY115/96-1* and *ShWRKY107/105*, respectively). Another 3-7 *ShWRKYs* were distributed on other chromosomes (Supplementary Figure 4). Gene duplication (tandem/segmental) events for *ShWRKY* genes are shown in Figure 4. Four gene pairs (*ShWRKY118-1* and *ShWRKY118-2*, *ShWRKY44-1* and *ShWRKY44-2*, *ShWRKY58* and *ShWRKY59-1*, *ShWRKY124-2* and *ShWRKY125-3*) displayed tandem duplication on their respective chromosomes, whereas 11 gene pairs were segmentally duplicated (Figure 4). Collinearity analysis was used to examine the evolutionary relationship of WRKY family genes among *S. spontaneum* AP85-441 and *Saccharum* spp. hybrid R570 revealed robust orthologs of *ShWRKY* genes (Supplementary Figure 5). For example, *ShWRKY42* displayed syntenic association with two *SsWRKY42* genes (Sspon.03G0003610 and Sspon.03G0029850) from AP85-441. Moreover, *ShWRKY29-1/61-3/69-1/96-1* also displayed a syntenic relationship with one different gene from AP85-441. Taken together, 101 *SsWRKY* genes from AP85-441 were lost in R570 (Supplementary Table 3).

Five miRNAs (ssp-miR159a, ssp-miR827, ssp-miR528, ssp-miR167-b, and ssp-miR444c-3p) were forecasted to target five *ShWRKY* genes (Supplementary Figure 6). Two miRNAs (ssp-miR159a and ssp-miR827) identified in the AP85-441 genome targeted one *ShWRKY107* gene, and ssp-miR528 targeted two *ShWRKY* genes (*ShWRKY136-1/72-2*). Moreover, ssp-miR167b targeted the *ShWRKY154-1* gene and ssp-miR444c-3p targeted *ShWRKY130*. Ka/Ks ratios calculated to analyze evolutionary relationships among *ShWRKYs* were mainly < 1, suggesting that they were under purifying selection, but ratios >1 determined for *ShWRKY118-1/118-2* indicated positive selection (Supplementary Table 4).

### Transcript Expression of *ShWRKY* Gene Responses to *Xanthomonas albilineans* Infection

The published RNA-seq dataset from two cultivars LCP85-384 and ROC20 trigged by *Xa* was used to assess *ShWRKY* gene expression patterns. Overall, three expression patterns were observed among 66 *ShWRKY* alleles. First, 26 *ShWRKY* alleles had irregular expression profiles in two cultivars at different time points (e.g., *ShWRKY49-3/52-1/93-3/138*). Second, transcript levels of 17 *ShWRKY* alleles (e.g., *ShWRKY45-1/54-1/69-1/69-1/130*) were significantly increased [>1.5 (log_2_FC)] in two cultivars across all time points. Third, transcript levels of 23 *ShWRKY* alleles were significantly increased in LCP85-384 but significantly decreased or unchanged in ROC20. For instance, transcript levels (log_2_FC) of *ShWRKY13-2/39-1/118-2/125-3* genes ranged from −5.46 to 0.58 in ROC20 but were between 1.38 and 7.16 in LCP85-384 at 24–72 hpi (Supplementary Figure 7).

**FIGURE 5:**
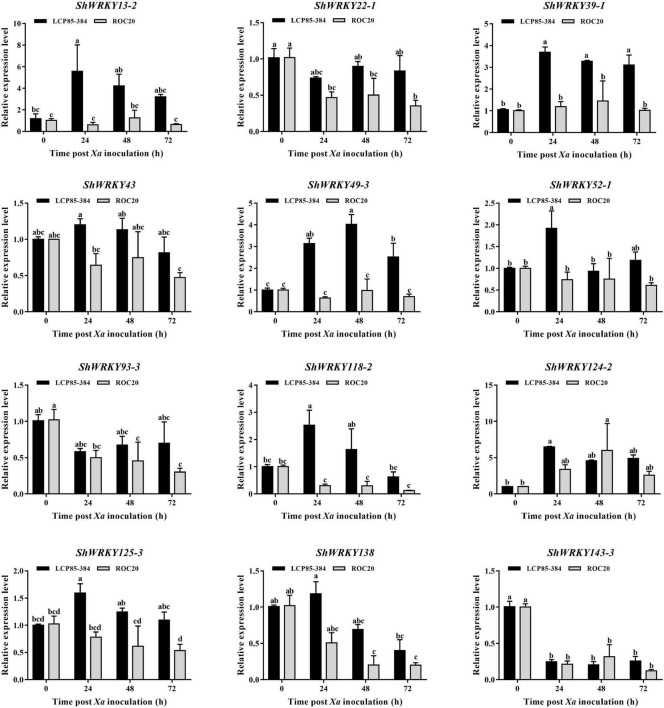
Expression profiling of twelve *ShWRKY* genes at different times post-*Xa* inoculation in LCP85-384 (black gars) and ROC20 (gray bars) cultivars. Values for relative expression levels are means ± standard errors. Means having the same letters above the vertical bars are not significantly different at a 5% level of probability.

To further assess the temporal expression patterns of *ShWRKY* genes in two cultivars in response to *Xa* infection, 12 candidate genes, *ShWRKY43/124-2/125-3* in *ShWRKY* group I, *ShWRKY52-1/93-3/138/143-3* in *ShWRKY* group II, and *ShWRKY13-2/22-1/39-1/49-3/118-2* in *ShWRKY* group III were selected for RT-qPCR assay. Compared to controls (0 hpi), four candidate genes (*ShWRKY13-2/39-1/49-3/125-3*) were significantly upregulated in LCP85-384 but were significantly downregulated or unchanged in ROC20 at 24–72 hpi. The transcript levels of *ShWRKY13-2/39-1/49-3* genes were increased by 3.7–5.6-fold in LCP85-384, while those for *ShWRKY125-3* were increased by 10–60% (Figure 5). Some *ShWRKY* genes, such as *ShWRKY43/52-1/118-2/138*, were only upregulated at a specific time point. The transcript levels of these four *ShWRKY* genes were increased by 1.2-, 1.9-, 2.5-, and 1.2-fold at 24 hpi, respectively. Notably, *ShWRKY124-2* was dramatically upregulated with an increase of >6.5-fold, whereas the expression of *ShWRKY22-1/93-3/143-3* was downregulated at 24–72 hpi in the two cultivars (Figure 5).

### Transcript Expression of *ShWRKY* Genes Responses to Nitrogen Implantation Dosage

**FIGURE 6:**
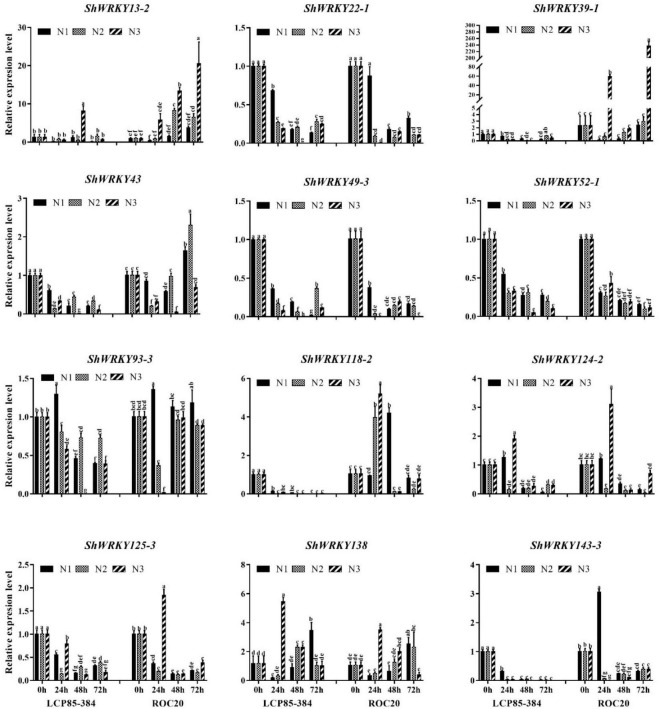
Expression profiling of twelve *ShWRKY* genes at different times after nitrogen implantation dosage in LCP85-384 and ROC20 cultivars. Values are means ± standard errors. Means having the same letters above the vertical bars are not significantly different at a 5% level of probability.

To characterize *ShWRKY* genes responding to different N input dosages in two cultivars, the transcriptional expression of the 12 above-mentioned *ShWRKY*s was examined by RT-qPCR. Transcript levels of three *ShWRKY* genes (*ShWRKY22-1/49-3/52-1*) were significantly downregulated in two cultivars in response to three N implantation dosages (N1, N2, and N3) across 24–72 h post-application sampling (hpas), while similar transcriptional expression trends were observed for two other *ShWRKY* genes (*ShWRKY125-3/143-3*) in all treatments except for N implantation dosages N3 and N1 in ROC20 at 24 hpas. These results suggested that these *ShWRKYs* played negative roles in the response of the two cultivars to N implantation dosages. However, transcript levels of four *ShWRKY* genes (*ShWRKY13-2/39-1/43/118-2*) were increased in ROC20, but decreased in LCP85-384 under the N implantation dosages, suggesting that these genes play different roles in the two cultivars in response to the extra N applications. Notably, the overall transcript expression of *ShWRKY138* was significantly upregulated in two cultivars at the highest input dosages at 24–72 hpas, suggesting that this gene is a positive regulator for the response of sugarcane to extra N applications. In addition, the expression patterns of some *ShWRKY* genes (e.g., *ShWRKY93-3* and *ShWRKY124-2*) differed between the two cultivars in response to N implantation dosage at different time points (Figure 6).

### Protein-Protein Interactions Among ShWRKYs

**FIGURE 7:**
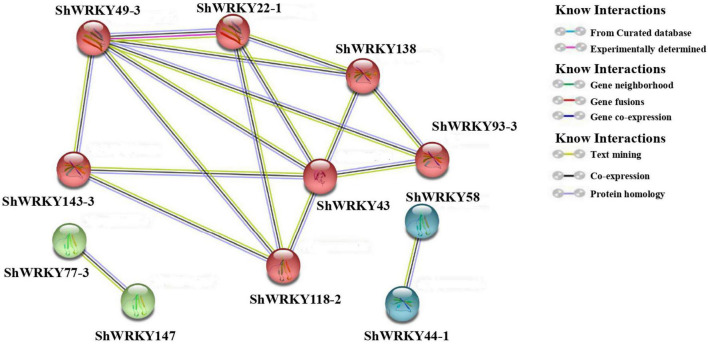
Predicted protein-protein interactions of *ShWRKYs* according to their orthologs in *A. thaliana*. Only those pairs with >30% sequence identity between *ShWRKYs* and *AtWRKYs* and an interaction score >0.8 are shown in the network. Line and node colors indicate different kinds and degrees of interactions, respectively. Ribbon diagrams are shown in each node.

Prediction of protein-protein interaction networking among ShWRKYs according to their orthologs in *A. thaliana* depicted the presence of strong interaction networking among seven ShWRKY proteins, namely, ShWRKY22-1, ShWRKY43, ShWRKY49-3, ShWRKY93-3, ShWRKY118-2, ShWRKY138, and ShWRKY143-3. ShWRKY43 and ShWRKY49-3 are core proteins that interacted with the other six ShWRKY proteins, followed by ShWRKY22 and ShWRKY138 which interacted with four other ShWRKY proteins. Point-to-point interactions were also observed between ShWRKY77-3 and ShWRKY147 as well as ShWRKY44-1 and ShWRKY58 (Figure 7). Detailed information about protein-protein interactions and functional annotations is given in Supplementary Table 5.

## Discussion

WRKY TFs are part of a diverse and versatile gene family that is found in many crop species with considerable diversity in terms of number, structure, and function (Li et al., 2020; Wani et al., 2021). The number of WRKY family members ranged from 42 genes in *Akebia trifoliata* (Wen et al., 2022) to 242 genes in *Camelina sativa* (Song et al., 2020). In this study, we showed that the *Saccharum* spp. hybrid R570 genome had 53 *ShWRKYs*, whereas *S. spontaneum* AP85-441 was previously shown to have 154 *SsWRKY* genes (Li et al., 2020). The number of *ShWRKYs* genes identified by us was far lower than the 94 genes in *Sorghum bicolor* (Baillo et al., 2020) and 140 genes in *Zea mays* (Hu et al., 2021). This reduction could be due to the BAC-based monoploid genome sequence of the R570 cultivar that was produced by exploiting the collinearity with sorghum and assembling a 382-Mb single tiling path of a high-quality sequence (25,316 protein-coding genes predicted) (Garsmeur et al., 2018). Meanwhile, the haploid *S. spontaneum* AP85-441 was assembled with a 2.9 Gbp genome bearing 35,525 genes with defined alleles (Zhang et al., 2018). Segmental duplication events of *ShWRKYs* were thus more than tandem duplication events in the R570 genome sequence. A similar evolutionary pattern for *SsWRKYs* was found in the AP85-441 genome (Li et al., 2020). Therefore, the high diversity/variability in the number of WRKYs among different plant species might be due to different evolutionary indices or duplication of entire genomes during the evolutionary phase. Specifically, the unique profiles of duplicated (tandem/segmental) genes following the duplication of whole genomes might also be responsible for long-term evolutionary transitions (Van De Peer et al., 2017). Overall, numerous studies also confirmed that segmental and tandem duplications, especially for the former events, might be key driving forces in the evolution and expansion of WRKYs in different crop plants (He et al., 2016; Xie et al., 2018).

In this study, high transcript levels of some *ShWRKYs* genes, such as (*ShWRKY13-2/39-1/52-1/118-2/125-3*), were observed in LCP85-384 at all or only specific time points, whereas those genes were found to be downregulated in ROC20 based on the transcriptome dataset and RT-qPCR assay. This result suggests that these genes might play a role in defenses against *Xa* infection. *Arabidopsis WRKY46* (homolog of *ShWRKY118-2*), *WRKY53* (homolog of *ShWRKY39-1* or *ShWRKY49-3*), and *WRKY70* (homolog of *ShWRKY13-2*) were previously shown to positively regulate basal defense responses against pathogen infection (Hu et al., 2012). More recently, Hu et al. (2021) also proposed that *BnMED16* confers resistance against *Sclerotinia sclerotiorum* by regulating *BnWRKY33* (homolog of *ShWRKY124-2* or *ShWRKY125-3*)-activated defense signaling and *BnMED25*-mediated defense pathways in *Brassica napus*. Chen et al. (2021) reported constitutive involvement of *WRKY70* (*homolog of ShWRKY13-2*) in defense responses against the bacterial pathogen *P. syringae* pv. *maculicola*. Interestingly, previous findings also supported the results of this study and revealed the involvement of *PtrWRKY73* (homolog of *ShWRKY43*) against disease resistance in *Arabidopsis* (Duan et al., 2015). Wen et al. (2022) reported the involvement of *AktWRKY33* (homolog of *ShWRKY124-2* or *ShWRKY125-3*) in *Akebia trifoliata* plant disease (caused by *Colletotrichum acutatum*) resistance.

WRKY TFs also play a pivotal role in diverse responses to both abiotic and biotic stresses as well as nutrient imbalances (Wani et al., 2021). In this study, most tested *ShWRKY* genes had a negative role in sugarcane cultivars in response to different N implantation dosages, but *ShWRKY138* had a positive role. Several *ShWRKY* genes were positively regulated in a particular cultivar under extra N supply. These results suggested that gene regulation of *ShWRKYs* depended on sugarcane genotypes that likely have diverse N use efficiency. Previous studies indeed demonstrated that WRKY responds to the N supply and metabolism. For example, 16 *JcWRKY* genes in *Jatropha curcas* responded to N starvation (Xiong et al., 2013). Some nitric oxide (NO)-responsive *AtWRKYs* were found in *A. thaliana* and *AtWRKY62* (homolog of *ShWRKY13-2*) and were shown to be involved in NO metabolism as evidenced by the negative role of the *atwrky62* mutant in plant growth; these plants showed significantly lower amounts of the NO donor S-Nitrosocysteine compared to wild-type plants (Imran et al., 2018). The *AtWRKY46* (homolog of *ShWRKY118-2*) in *A. thaliana* was shown to inhibit ammonium (NH_4_^+^) efflux by directly binding to the promoters of genes involved in GDP-D-mannose pyrophosphohydrolase (NUDX9) and indole acetic acid (IAA) conjugation (Di et al., 2021). However, few studies had examined the responses and functions of WRKY family genes relative to N implantation dosage in different crop plants.

Interestingly, the results of this study indicated that some *ShWRKY* genes (e.g., *ShWRKY13-2/39-1/43/49-3/52-1/118-2/125-3*) exhibited positive regulation in the LCP85-384 cultivar (resistant to leaf scald) in response to *Xa* infection, whereas others had negative regulation in response to different N implantation dosages. Our results suggested that extra N supply in plants likely affects resistance to leaf scald. The *ShWRKY43/49-3/52-1* genes were predicted to play crucial roles in the interaction network of ShWRKY proteins and could coordinate important crosstalk between defense responses to bacterial infection and extra N applications in sugarcane. High N dosages were associated with increased disease incidence (Shrivastava and Srivastava, 2012) and affected lateral roots development (O’Brien et al., 2016). Recently, Poll et al. (2020) suggested that reduced N dosage and septoria leaf blotch disease caused by *Zymoseptoria tritici* pathogen co-dependently altered expression of wheat WRKY TFs, and demonstrated that *WRKY68a* (homolog of *ShWRKY11*) may mediate a link between N dosage and increased tolerance to pathogen infection.

## Conclusion

In this study, 53 WRKY family genes with 66 alleles were systematically explored in the *Saccharum* spp. hybrid R570 and further classified into four main groups. The gene duplication and collinearity analysis provided valuable information about the evolutionary history of *ShWRKY* genes. Further, the RNA-seq dataset and/or RT-qPCR analysis suggested that *ShWRKY* genes may play a pivotal role in response to bacterial pathogen infection and N implantation dosage. Overall, systematic analysis of *ShWRKY* genes in sugarcane provides a basis for functional characterization in response to coupling effects of bacterial pathogen infection and different N levels. However, understanding the detailed mechanism of how *ShWRKY* genes regulate diverse biological functions in sugarcane that are involved in pathogen-triggered immunity and N-triggered response requires additional investigation.

## Data Availability Statement

The raw data supporting the conclusions of this article will be made available by the authors, without undue reservation.

## Author Contributions

TJ and S-JG conceptualized the study and contributed to writing, reviewing, and editing of the manuscript. S-JG contributed to resources. TJ, J-RZ, JL, and Z-TH contributed to writing the original draft. Q-NW and S-JG contributed to supervision, funding acquisition, and project administration. All authors have read and agreed to the final version of the manuscript.

## Conflict of Interest

The authors declare that the research was conducted in the absence of any commercial or financial relationships that could be construed as a potential conflict of interest.

## Publisher’s Note

All claims expressed in this article are solely those of the authors and do not necessarily represent those of their affiliated organizations, or those of the publisher, the editors and the reviewers. Any product that may be evaluated in this article, or claim that may be made by its manufacturer, is not guaranteed or endorsed by the publisher.

## Footnotes

1
http://sugarcane-genome.cirad.fr/

2
http://blast.ncbi.nlm.nih.gov/

3
http://xfam.org/

4
http://smart.emblheidelberg.de/

5
http://web.expasy.org/compute_pi/

6
http://cello.life.nctu.edu.tw/cello2go/

7
http://www.string-db.org

8
http://gsds.cbi.pku.edu.cn/

9
http://meme-suite.org/index.html

10
http://www.dna.affrc.go.jp/PLACE/

11
https://phytozome-next.jgi.doe.gov/info/SbicolorSC187_v1_1

12
https://github.com/tangerzhang/FAFUcgb/tree/master/easy_KaKs

13
https://www.genscript.com/tools/real-time-pcr-taqman-primer-design-tool

14
https://www.statistix.com/
