# Supplementary material for: Identification and Expression Profiling of WRKY Family Genes in Sugarcane in Response to Bacterial Pathogen Infection and Nitrogen Implantation Dosage
Source: Front Plant Sci. 2022 Jun 9;13:917953. doi: 10.3389/fpls.2022.917953 (PMC9218642; doi:10.3389/fpls.2022.917953)
Supplement: Supplementary file 1 [file Data_Sheet_1.ZIP › 2 Supplementry file-20220430.docx]

**Table S1** Primers sequences used for RT-qPCR analysis.

| **Gene ID** | **Transcript ID** | **Forward Primer** | **Reverse Primer** |
| --- | --- | --- | --- |
| ShWRKY13-2 | Cluster-4871.377471 | CCACAACACGACATCCTTCC | AGGCCAGAGGAGGTAGTACA |
| ShWRKY22-1 | Cluster-4871.272134 | TGCTCATCACCACCTCCAAC | CCCTCACCTCATGCCTCTTC |
| ShWRKY39-1 | Cluster-4871.272138 | GCTTGGCTCCAAGAATCTCC | GCCGTTCAAGAACACCAAGA |
| ShWRKY43 | Cluster-4871.279230 | CATGAGCGACATCGACATCC | CGTCGTGCACTTGTAGTAGC |
| ShWRKY49-3 | Cluster-4871.239438 | AGATGCAAGCACGTTTGTGT | ATTTCATGTTCCCGCTCGAC |
| ShWRKY52-1 | Cluster-4871.266283 | GTCGCTGGAGTAGAGGACGA | GCCATCCCATCAGGAGAGGT |
| ShWRKY93-3 | Cluster-4871.201784 | GCTTCCCGGAGGACATCA | GGACTTCACGAAGCCGAATC |
| ShWRKY118-2 | Cluster-5862.1 | CCTTTGCCGAACTCCCAGTC | TGCAATGCGGCTGAGTTCAT |
| ShWRKY124-2 | Cluster-4871.279239 | TACACCGACTTCACGTTCCA | CGGATGACTGCGGAAGAAAG |
| ShWRKY125-3 | Cluster-4871.191842 | CCTGCTCCACTATTCCGCTA | CTGTCGTCTTGCTGAGATGC |
| ShWRKY138 | Cluster-51923.1 | ATCACCACCGTCGTGTCC | CGTACTACCGCTGCACCAT |
| ShWRKY143-3 | Cluster-4871.122438 | GAGTGCAAGCCCAAGGTG | ACCTTCTGACCGTACTTCCG |

| **Table S2** Physio-chemical properties of *ShWRKYs*. | | | | | | | | | | | | |
| --- | --- | --- | --- | --- | --- | --- | --- | --- | --- | --- | --- | --- |
| **Query ID** | **Subject ID** | **Nomenclature** | **Group** | **Conserved motif** | **Zinc finger type** | **Amino acid length (aa)** | **MW (kDa)** | **PI** | **II** | **AI** | **GRAVY** | **Subcellular localization** |
| **Sspon.001A0015280** | **Sh_208G02_contig-1_p000070** | **ShWRKY2-1** | **I** | **WRKYGQK** | **C-X4-C-X23-HSH(C)** | **491** | **52024.03** | **6.84** | **64.27** | **56.17** | **-0.757** | **Nucleus** |
|  |  |  |  | **WRKYGQK** | **C-X4-C-X23-HNH(N)** |  |  |  |  |  |  |  |
| **Sspon.002C0007220** | **Sh_231B08_p000100** | **ShWRKY27-3** | **I** | **WRKYGQK** | **C-X4-C-X23-HNH(C)** | **574** | **61769.12** | **5.99** | **51.48** | **54.06** | **-0.822** | **Nucleus** |
|  |  |  |  | **WRKYGQK** | **C-X4-C-X23-HNH(N)** |  |  |  |  |  |  |  |
| **Sspon.002D0038610** | **Sh_243J22_p000120** | **ShWRKY40-2** | **I** | **WRKYGQK** | **C-X4-C-X23-HNH(C)** | **489** | **51664.72** | **6.93** | **54.28** | **54.42** | **-0.755** | **Nucleus** |
|  |  |  |  | **WRKYGQK** | **C-X4-C-X23-HNH(N)** |  |  |  |  |  |  |  |
| **Sspon.003B0008770** | **Sh_027M21_p000160** | **ShWRKY42-2** | **I** | **WRKYGQK** | **C-X4-C-X23-HNH(C)** | **375** | **40601.32** | **8.21** | **61.19** | **40.69** | **-0.951** | **Nucleus** |
|  |  |  |  | **WRKYGQK** | **C-X4-C-X23-HNH(N)** |  |  |  |  |  |  |  |
| **Sspon.003D0007480** | **Sh_242F09_p000080** | **ShWRKY42-3** | **I** | **WRKYGQK** | **C-X4-C-X23-HNH(C)** | **544** | **58240.53** | **6.2** | **63.55** | **41.76** | **-0.799** | **Nucleus** |
|  |  |  |  | **WRKYGQK** | **C-X4-C-X23-HNH(N)** |  |  |  |  |  |  |  |
| **Sspon.003A0004330** | **Sh_214N15_p000020** | **ShWRKY43** | **I** | **WRKYGQK** | **C-X4-C-X23-HNH(C)** | **554** | **59104.45** | **6.2** | **61.43** | **41.90** | **-0.78** | **Nucleus** |
|  |  |  |  | **WRKYGQK** | **C-X4-C-X23-HNH(N)** |  |  |  |  |  |  |  |
| **Sspon.005B0009940** | **Sh_239F16_p000010** | **ShWRKY82-2** | **I** | **WRKYGQK** | **C-X4-C-X23-HNH(C)** | **721** | **76738.43** | **6.66** | **49.39** | **58.22** | **-0.661** | **Nucleus** |
|  |  |  |  | **WRKYGQK** | **C-X4-C-X23-HNH(N)** |  |  |  |  |  |  |  |
| **Sspon.006B0010240** | **Sh_213P14_p000040** | **ShWRKY105** | **I** | **WRKYGQK** | **C-X4-C-X23-HNH(C)** | **395** | **43360.31** | **8.01** | **57.43** | **60.20** | **-0.765** | **Nucleus** |
|  |  |  |  | **WRKYGQK** | **C-X4-C-X23-HNH(N)** |  |  |  |  |  |  |  |
| **Sspon.007B0007050** | **Sh_250M05_p000080** | **ShWRKY124-1** | **I** | **WRKYGQK** | **C-X4-C-X23-HNH(C)** | **565** | **59558.24** | **6.21** | **47.37** | **48.11** | **-0.623** | **Nucleus** |
|  |  |  |  | **WRKYGQK** | **C-X4-C-X23-HNH(N)** |  |  |  |  |  |  |  |
| **Sspon.007C0005220** | **Sh_203B03_p000030** | **ShWRKY124-2** | **I** | **WRKYGQK** | **C-X4-C-X23-HNH(C)** | **566** | **59733.49** | **6.28** | **47.37** | **47.84** | **-0.614** | **Nucleus** |
|  |  |  |  | **WRKYGQK** | **C-X4-C-X23-HNH(N)** |  |  |  |  |  |  |  |
| **Sspon.007C0011650** | **Sh_240H04_p000010** | **ShWRKY125-3** | **I** | **WRKYGQK** | **C-X4-C-X23-HNH(C)** | **560** | **59214.7** | **9.17** | **56.87** | **58.50** | **-0.635** | **Nucleus** |
|  |  |  |  | **WRKYGQK** | **C-X4-C-X23-HNH(N)** |  |  |  |  |  |  |  |
| **Sspon.008C0006550** | **Sh_254F21_p000020** | **ShWRKY143-3** | **II a** | **WRKYGQK** | **C-X5-C-X23-HNH** | **333** | **35632.31** | **9.58** | **57.06** | **70.18** | **-0.527** | **Nucleus** |
| **Sspon.008B0006380** | **Sh_254F21_p000050** | **ShWRKY145-2** | **II a** | **WRKYGQK** | **C-X5-C-X23-HNH** | **346** | **37301.14** | **9.56** | **55.85** | **71.18** | **-0.566** | **Nucleus** |
| **Sspon.003A0019860** | **Sh_201A18_p000010** | **ShWRKY52-1** | **II b** | **WRKYGQK** | **C-X5-C-X23-HNH** | **562** | **58391.03** | **6.37** | **51.85** | **66.74** | **-0.417** | **Nucleus** |
| **Sspon.003A0028210** | **Sh_223B05_p000020** | **ShWRKY54-1** | **II b** | **WRKYGQK** | **C-X5-C-X23-HNH** | **533** | **54807.06** | **6.95** | **47.46** | **66.15** | **-0.355** | **Nucleus** |
| **Sspon.003A0015220** | **Sh_222N02_contig-1_p000010** | **ShWRKY64-1** | **II b** | **WRKYGQK** | **C-X5-C-X23-HNH** | **341** | **35228.77** | **9.14** | **49** | **72.84** | **-0.216** | **Nucleus** |
| **Sspon.003C0038970** | **Sh_216M23_contig-1_p000060** | **ShWRKY68-3** | **II b** | **WRKYGQK** | **C-X5-C-X23-HNH** | **579** | **60490.45** | **9.02** | **50.75** | **63.73** | **-0.495** | **Nucleus** |
| **Sspon.003D0028050** | **Sh_226P18_p000030** | **ShWRKY68-4** | **II b** | **WRKYGQK** | **C-X5-C-X23-HNH** | **608** | **63797.05** | **8.06** | **53.66** | **61.63** | **-0.512** | **Nucleus** |
| **Sspon.004C0003790** | **Sh_242K18_contig-1_p000020** | **ShWRKY77-2** | **II b** | **WRKYGQK** | **C-X5-C-X23-HNH** | **578** | **61136.26** | **5.36** | **49.1** | **54.91** | **-0.765** | **Nucleus** |
| **Sspon.004D0004630** | **Sh_245H10_contig-2_p000030** | **ShWRKY77-3** | **II b** | **WRKYGQK** | **C-X5-C-X23-HNH** | **579** | **61277.61** | **5.27** | **50.06** | **56.68** | **-0.707** | **Nucleus** |
| **Sspon.007C0017400** | **Sh_240J24_p000140** | **ShWRKY130** | **II b** | **WRKYGQK** | **C-X5-C-X23-HNH** | **540** | **55661.07** | **5.81** | **55.59** | **63.43** | **-0.388** | **Nucleus** |
| **Sspon.007D0002070** | **Sh_249K07_p000070** | **ShWRKY138** | **II b** | **WRKYGQK** | **C-X5-C-X23-HNH** | **380** | **38740.36** | **7.13** | **51.62** | **58.03** | **-0.366** | **Nucleus** |
| **Sspon.008B0019640** | **Sh_238H15_contig-1_p000040** | **ShWRKY147** | **II b** | **WRKYGQK** | **C-X5-C-X23-HNH** | **573** | **59561.2** | **8.48** | **42.78** | **62.86** | **-0.436** | **Nucleus** |
| **Sspon.002B0013050** | **Sh_201J12_contig-1_p000130** | **ShWRKY29-1** | **II c** | **WRKYGQK** | **C-X4-C-X23-HNH** | **584** | **62330.02** | **6.1** | **59.71** | **57.5** | **-0.567** | **Nucleus** |
| **Sspon.002A0015520** | **Sh_202L17_p000170** | **ShWRKY29-2** | **II c** | **WRKYGQK** | **C-X4-C-X23-HNH** | **584** | **62459.18** | **6.07** | **59.63** | **56.66** | **-0.567** | **Nucleus** |
| **Sspon.003C0003070** | **Sh_052F15_p000050** | **ShWRKY55** | **II c** | **WRKYGQK** | **C-X4-C-X23-HLH** | **362** | **39502.33** | **4.72** | **74.54** | **67.43** | **-0.537** | **Nucleus** |
| **Sspon.003D0013750** | **Sh_226A20_p000060** | **ShWRKY61-3** | **II c** | **WRKYGQK** | **C-X4-C-X23-HTH** | **393** | **40781.54** | **7.75** | **58.82** | **50.76** | **-0.583** | **Nucleus** |
| **Sspon.003D0015740** | **Sh_244I15_p000010** | **ShWRKY72-2** | **II c** | **WRKYGQK** | **C-X4-C-X23-HTH** | **361** | **39104.18** | **6.57** | **61.99** | **43.60** | **-0.909** | **Nucleus** |
| **Sspon.005A0007770** | **Sh_228G23_p000070** | **ShWRKY81-1** | **II c** | **WRKYGQK** | **C-X4-C-X23-HTH** | **249** | **27131.85** | **8.82** | **42.3** | **62.05** | **-0.476** | **Nucleus** |
| **Sspon.007A0001520** | **Sh_253C05_p000050** | **ShWRKY116-1** | **II c** | **WRKYGQK** | **C-X4-C-X23-HTH** | **362** | **37938.05** | **6.01** | **57.58** | **62.85** | **-0.562** | **Nucleus** |
| **Sspon.007B0013420** | **Sh_243J21_p000030** | **ShWRKY126** | **II c** | **WRKYGQK** | **C-X4-C-X23-HLH** | **359** | **39205.95** | **4.68** | **71.62** | **69.05** | **-0.501** | **Nucleus** |
| **Sspon.007C0001580** | **Sh_247E21_p000130** | **ShWRKY127-1** | **II c** | **WRKYGQK** | **C-X4-C-X23-HTH** | **426** | **44902.86** | **6.34** | **41.74** | **54.23** | **-0.543** | **Nucleus** |
| **Sspon.001B0042840** | **Sh_220B19_contig-2_p000040** | **ShWRKY6** | **II d** | **WRKYGQK** | **C-X5-C-X23-HNH** | **420** | **44765.05** | **9.33** | **60.54** | **64.19** | **-0.427** | **Nucleus** |
| **Sspon.001A0029570** | **Sh_248B23_p000040** | **ShWRKY15-1** | **II d** | **WRKYGQK** | **C-X5-C-X23-HNH** | **351** | **37885.78** | **9.72** | **48.92** | **64.44** | **-0.572** | **Nucleus** |
| **Sspon.001C0028410** | **Sh_234E24_p000010** | **ShWRKY15-3** | **II d** | **WRKYGQK** | **C-X5-C-X23-HNH** | **291** | **31879.28** | **9.92** | **49.77** | **62.65** | **-0.583** | **Nucleus** |
| **Sspon.002C0039810** | **Sh_251N22_p000020** | **ShWRKY38-2** | **II d** | **WRKYGQK** | **C-X5-C-X23-HNH** | **371** | **39448.87** | **10.01** | **55.37** | **64.02** | **-0.511** | **Nucleus** |
| **Sspon.005D0007330** | **Sh_219N16_p000090** | **ShWRKY93-3** | **II d** | **WRKYGQK** | **C-X5-C-X23-HRH** | **321** | **33758.37** | **9.96** | **49.27** | **70.06** | **-0.347** | **Nucleus** |
| **Sspon.ctg0653120** | **Sh_208J20_p000050** | **ShWRKY154-1** | **II d** | **WRKYGQK** | **C-X5-C-X23-HNH** | **401** | **43213.29** | **10.08** | **55.36** | **69.35** | **-0.504** | **Nucleus** |
| **Sspon.ctg0653120** | **Sh_217N06_contig-1_p000050** | **ShWRKY154-2** | **II d** | **WRKYGQK** | **C-X5-C-X23-HNH** | **402** | **43388.53** | **10.18** | **58.1** | **69.18** | **-0.517** | **Nucleus** |
| **Sspon.001D0048240** | **Sh_221G17_p000010** | **ShWRKY3-2** | **II e** | **WRKYGQK** | **C-X5-C-X23-HDH** | **392** | **42254.28** | **6.21** | **60.77** | **57.37** | **-0.532** | **Nucleus** |
| **Sspon.003A0007880** | **Sh_234G14_p000150** | **ShWRKY45-1** | **II e** | **WRKYGQK** | **C-X5-C-X23-HNH** | **312** | **33770.13** | **4.8** | **85.57** | **51.44** | **-0.894** | **Nucleus** |
| **Sspon.003C0016500** | **Sh_225K23_p000010** | **ShWRKY69-1** | **II e** | **WRKYGQK** | **C-X5-C-X23-HSH** | **309** | **31329.62** | **4.98** | **57.79** | **55.73** | **-0.336** | **Chloroplast** |
| **Sspon.003D0011390** | **Sh_008F19_p000010** | **ShWRKY69-2** | **II e** | **WRKYGQK** | **C-X5-C-X23-HSH** | **309** | **31344.63** | **5.14** | **57.34** | **55.40** | **-0.332** | **Chloroplast** |
| **Sspon.004B0005840** | **Sh_245J18_p000030** | **ShWRKY74** | **II e** | **WRKYGQK** | **C-X5-C-X23-HNH** | **483** | **50537.28** | **5.72** | **50.92** | **55.07** | **-0.467** | **Nucleus** |
| **Sspon.004A0017370** | **Sh_240N09_p000010** | **ShWRKY75-1** | **II e** | **WRKYGQK** | **C-X5-C-X23-HNH** | **373** | **39653.37** | **5.95** | **75.42** | **56.81** | **-0.696** | **Nucleus** |
| **Sspon.004C0017350** | **Sh_014J13_p000020** | **ShWRKY75-3** | **II e** | **WRKYGQK** | **C-X5-C-X23-HNH** | **391** | **42166.28** | **6.52** | **79.81** | **53.20** | **-0.792** | **Nucleus** |
| **Sspon.007C0000760** | **Sh_253C05_p000110** | **ShWRKY136-1** | **II e** | **WRKYGQK** | **C-X5-C-X23-HCH** | **341** | **36544.73** | **6.07** | **52.71** | **66.19** | **-0.537** | **Nucleus** |
| **Sspon.001D0012080** | **Sh_205I06_p000030** | **ShWRKY13-2** | **III** | **WRKYGQK** | **C-X7-C-X23-HTC** | **326** | **33922.32** | **5.97** | **45.28** | **68.65** | **-0.379** | **Nucleus** |
| **Sspon.002C0021640** | **Sh_251G09_p000010** | **ShWRKY19-2** | **III** | **WRKYGQK** | **C-X7-C-X23-HTC** | **330** | **35100.91** | **5.53** | **55.47** | **68.88** | **-0.323** | **Nucleus** |
| **Sspon.002D0017270** | **Sh_251G09_p000020** | **ShWRKY19-3** | **III** | **WRKYGQK** | **C-X7-C-X23-HTC** | **310** | **33533.57** | **6.82** | **47.48** | **70.48** | **-0.387** | **Nucleus** |
| **Sspon.002A0022890** | **Sh_236L15_p000040** | **ShWRKY22-1** | **III** | **WRKYGQK** | **C-X7-C-X23-HTC** | **296** | **31734.37** | **5.87** | **48.06** | **73.51** | **-0.34** | **Nucleus** |
| **Sspon.002A0022890** | **Sh_236L15_p000030** | **ShWRKY22-2** | **III** | **WRKYGQK** | **C-X7-C-X23-HTC** | **296** | **31723.37** | **5.75** | **48.34** | **75.81** | **-0.341** | **Nucleus** |
| **Sspon.002B0020260** | **Sh_236L15_p000020** | **ShWRKY39-1** | **III** | **WRKYGQK** | **C-X7-C-X23-HTC** | **318** | **33664.37** | **5.88** | **45.95** | **72.17** | **-0.416** | **Nucleus** |
| **Sspon.003A0004520** | **Sh_252H06_contig-2_p000060** | **ShWRKY44-1** | **III** | **WRKYGQK** | **C-X7-C-X23-HTC** | **321** | **35051.99** | **5.44** | **65.44** | **60.31** | **-0.607** | **Nucleus** |
| **Sspon.003C0003120** | **Sh_252H06_contig-1_p000030** | **ShWRKY44-2** | **III** | **WRKYGQK** | **C-X7-C-X23-HTC** | **321** | **35051.99** | **5.44** | **65.44** | **60.31** | **-0.607** | **Nucleus** |
| **Sspon.003C0019610** | **Sh_247G22_p000020** | **ShWRKY49-3** | **III** | **WRKYGQK** | **C-X7-C-X23-HTC** | **343** | **35555.72** | **7.03** | **51.63** | **58.40** | **-0.362** | **Nucleus** |
| **Sspon.003C0011790** | **Sh_252H06_contig-1_p000050** | **ShWRKY58** | **III** | **WRKYGKK** | **C-X7-C-X23-HTC** | **366** | **40002.88** | **6.09** | **37.76** | **65.36** | **-0.455** | **Nucleus** |
| **Sspon.003A0004530** | **Sh_252H06_contig-1_p000060** | **ShWRKY59-1** | **III** | **WRKYGQK** | **C-X7-C-X23-HTC** | **261** | **29158.9** | **5.83** | **66.53** | **39.69** | **-0.944** | **Nucleus** |
| **Sspon.005A0027240** | **Sh_212A12_p000140** | **ShWRKY96-1** | **III** | **WRKYGEK** | **C-X7-C-X23-HTC** | **350** | **37872.72** | **5.77** | **53.9** | **69.00** | **-0.399** | **Nucleus** |
| **Sspon.006D0021290** | **Sh_214M05_p000080** | **ShWRKY115** | **III** | **WRKYGEK** | **C-X7-C-X23-HTC** | **315** | **35665.85** | **5.17** | **47.16** | **66.86** | **-0.605** | **Nucleus** |
| **Sspon.007A0009860** | **Sh_222I14_contig-2_p000020** | **ShWRKY118-1** | **III** | **WRKYGQK** | **C-X7-C-X23-HTC** | **310** | **34112.27** | **5** | **61.86** | **57.39** | **-0.504** | **Nucleus** |
| **Sspon.007A0009860** | **Sh_222I14_contig-4_p000010** | **ShWRKY118-2** | **III** | **WRKYGQK** | **C-X7-C-X23-HTC** | **303** | **33308.44** | **5.38** | **60.95** | **59.01** | **-0.535** | **Nucleus** |
| **Sspon.007D0024420** | **Sh_230O01_p000020** | **ShWRKY131-3** | **III** | **WRKYGQK** | **C-X7-C-X23-HIC** | **269** | **30371.18** | **6.26** | **42.76** | **77.17** | **-0.548** | **Nucleus** |
| **Sspon.007C0025650** | **Sh_235I18_p000080** | **ShWRKY132-2** | **III** | **WRKYGQK** | **C-X7-C-X23-HIC** | **265** | **29935.82** | **6.26** | **45.21** | **82.00** | **-0.524** | **Nucleus** |
| **Sspon.008A0018670** | **Sh_248N23_p000020** | **ShWRKY146-1** | **III** | **WRKYGQK** | **C-X7-C-X23-HTC** | **363** | **39324.53** | **6.01** | **56.49** | **53.66** | **-0.657** | **Nucleus** |
| **Sspon.006B0012170** | **Sh_205K03_p000030** | **ShWRKY107** | **IV** | **WRKYLWL** | **-** | **843** | **95533.93** | **6.07** | **54.77** | **79.16** | **-0.464** | **Nucleus** |

**Table S3** Collinearity analysis to appraise the evolutionary relationship of WRKY family genes among *S. spontaneum* (AP85-441) and *Saccharum* spp. hybrid R570.

| **Highlighted genes in up-genome** | | | | |
| --- | --- | --- | --- | --- |
| **Chr No.** | ***S. spont* IDs** |  | **Chr No.** | **R570 IDs** |
| Chr2A | Sspon.02G0012100-1A | == | Sh_201J12_contig-1 | Sh_201J12_contig-1_p000130 |
| Chr2A | Sspon.02G0012100-1A | == | Sh_202L17 | Sh_202L17_p000170 |
| Chr2A | Sspon.02G0027610-1A | == | Sh_243J22 | Sh_243J22_p000120 |
| Chr2B | Sspon.02G0012100-2B | == | Sh_202L17 | Sh_202L17_p000170 |
| Chr2D | Sspon.02G0027610-2D | == | Sh_243J22 | Sh_243J22_p000120 |
| Chr3A | Sspon.03G0003610-1A | == | Sh_027M21 | Sh_027M21_p000160 |
| Chr3A | Sspon.03G0003610-1A | == | Sh_214N15 | Sh_214N15_p000020 |
| Chr3A | Sspon.03G0007170-1A | == | Sh_225K23 | Sh_225K23_p000010 |
| Chr3A | Sspon.03G0009520-1A | == | Sh_226A20 | Sh_226A20_p000060 |
| Chr3B | Sspon.03G0029850-1B | == | Sh_027M21 | Sh_027M21_p000160 |
| Chr3B | Sspon.03G0029850-1B | == | Sh_214N15 | Sh_214N15_p000020 |
| Chr3C | Sspon.03G0029850-2C | == | Sh_027M21 | Sh_027M21_p000160 |
| Chr3C | Sspon.03G0029850-2C | == | Sh_214N15 | Sh_214N15_p000020 |
| Chr3C | Sspon.03G0025350-3C | == | Sh_216M23_contig-1 | Sh_216M23_contig-1_p000060 |
| Chr3C | Sspon.03G0029850-2C | == | Sh_242F09 | Sh_242F09_p000080 |
| Chr3D | Sspon.03G0029850-3D | == | Sh_027M21 | Sh_027M21_p000160 |
| Chr3D | Sspon.03G0029850-3D | == | Sh_214N15 | Sh_214N15_p000020 |
| Chr3D | Sspon.03G0025350-4D | == | Sh_216M23_contig-1 | Sh_216M23_contig-1_p000060 |
| Chr3D | Sspon.03G0007170-3D | == | Sh_225K23 | Sh_225K23_p000010 |
| Chr5A | Sspon.05G0009310-1A | == | Sh_239F16 | Sh_239F16_p000010 |
| Chr5C | Sspon.05G0021100-2C | == | Sh_212A12 | Sh_212A12_p000140 |
| Chr5D | Sspon.05G0009310-4D | == | Sh_239F16 | Sh_239F16_p000010 |
| Chr7C | Sspon.03G0029850-1P | == | Sh_027M21 | Sh_027M21_p000160 |
| Chr7C | Sspon.03G0029850-1P | == | Sh_203B03 | Sh_203B03_p000030 |
| Chr7C | Sspon.03G0029850-1P | == | Sh_214N15 | Sh_214N15_p000020 |
| Chr7C | Sspon.03G0009520-1P | == | Sh_247E21 | Sh_247E21_p000130 |
| Chr7C | Sspon.03G0029850-1P | == | Sh_250M05 | Sh_250M05_p000080 |
| Chr7D | Sspon.03G0029850-2P | == | Sh_027M21 | Sh_027M21_p000160 |
| Chr7D | Sspon.03G0029850-2P | == | Sh_203B03 | Sh_203B03_p000030 |
| Chr7D | Sspon.03G0029850-2P | == | Sh_214N15 | Sh_214N15_p000020 |
| Chr7D | Sspon.03G0029850-2P | == | Sh_250M05 | Sh_250M05_p000080 |
| **Highlighted genes in down-genome** | | | | |
| **Chr No.** | **R570 IDs** |  | **Chr No.** | ***S. spont* IDs** |
| Sh_027M21 | Sh_027M21_p000160 | == | Chr3A | Sspon.03G0003610-1A |
| Sh_027M21 | Sh_027M21_p000160 | == | Chr3B | Sspon.03G0029850-1B |
| Sh_027M21 | Sh_027M21_p000160 | == | Chr3C | Sspon.03G0029850-2C |
| Sh_027M21 | Sh_027M21_p000160 | == | Chr3D | Sspon.03G0029850-3D |
| Sh_027M21 | Sh_027M21_p000160 | == | Chr7C | Sspon.03G0029850-1P |
| Sh_027M21 | Sh_027M21_p000160 | == | Chr7D | Sspon.03G0029850-2P |
| Sh_203B03 | Sh_203B03_p000030 | == | Chr7C | Sspon.03G0029850-1P |
| Sh_203B03 | Sh_203B03_p000030 | == | Chr7D | Sspon.03G0029850-2P |
| Sh_239F16 | Sh_239F16_p000010 | == | Chr5A | Sspon.05G0009310-1A |
| Sh_239F16 | Sh_239F16_p000010 | == | Chr5D | Sspon.05G0009310-4D |
| Sh_226A20 | Sh_226A20_p000060 | == | Chr3A | Sspon.03G0009520-1A |
| Sh_250M05 | Sh_250M05_p000080 | == | Chr7C | Sspon.03G0029850-1P |
| Sh_250M05 | Sh_250M05_p000080 | == | Chr7D | Sspon.03G0029850-2P |
| Sh_225K23 | Sh_225K23_p000010 | == | Chr3A | Sspon.03G0007170-1A |
| Sh_225K23 | Sh_225K23_p000010 | == | Chr3D | Sspon.03G0007170-3D |
| Sh_243J22 | Sh_243J22_p000120 | == | Chr2A | Sspon.02G0027610-1A |
| Sh_243J22 | Sh_243J22_p000120 | == | Chr2D | Sspon.02G0027610-2D |
| Sh_212A12 | Sh_212A12_p000140 | == | Chr5C | Sspon.05G0021100-2C |
| Sh_216M23_contig-1 | Sh_216M23_contig-1_p000060 | == | Chr3C | Sspon.03G0025350-3C |
| Sh_216M23_contig-1 | Sh_216M23_contig-1_p000060 | == | Chr3D | Sspon.03G0025350-4D |
| Sh_247E21 | Sh_247E21_p000130 | == | Chr7C | Sspon.03G0009520-1P |
| Sh_201J12_contig-1 | Sh_201J12_contig-1_p000130 | == | Chr2A | Sspon.02G0012100-1A |
| Sh_242F09 | Sh_242F09_p000080 | == | Chr3C | Sspon.03G0029850-2C |
| Sh_214N15 | Sh_214N15_p000020 | == | Chr3A | Sspon.03G0003610-1A |
| Sh_214N15 | Sh_214N15_p000020 | == | Chr3B | Sspon.03G0029850-1B |
| Sh_214N15 | Sh_214N15_p000020 | == | Chr3C | Sspon.03G0029850-2C |
| Sh_214N15 | Sh_214N15_p000020 | == | Chr3D | Sspon.03G0029850-3D |
| Sh_214N15 | Sh_214N15_p000020 | == | Chr7C | Sspon.03G0029850-1P |
| Sh_214N15 | Sh_214N15_p000020 | == | Chr7D | Sspon.03G0029850-2P |
| Sh_202L17 | Sh_202L17_p000170 | == | Chr2A | Sspon.02G0012100-1A |
| Sh_202L17 | Sh_202L17_p000170 | == | Chr2B | Sspon.02G0012100-2B |

**Table S4** The non-synonymous (Ka) and synonymous (Ks) substitution ratios of *ShWRKYs*.

| **Gene 1** | **Gene 2** | **Ka** | **Ks** | **Ka/Ks** | **Selection Pressure** | **T=Ks/2r** | **T (MYa)** | **Divergence time** |
| --- | --- | --- | --- | --- | --- | --- | --- | --- |
| ShWRKY118-1 | ShWRKY118-2 | 0.013118 | 0.009542872 | 1.374667296 | Positive | 0.782202646 | 0.782202646 | 0.78 |
| ShWRKY44-1 | ShWRKY44-2 | 0 | 0 | 0 | 0 | 0 | 0 | 0.00 |
| ShWRKY59-1 | ShWRKY58 | 0.76749 | 1.226832266 | 0.625587058 | Purifying | 100.5600218 | 100.5600218 | 100.56 |
| ShWRKY125-3 | ShWRKY124-2 | 0.423626 | 0.723412595 | 0.585594499 | Purifying | 59.29611431 | 59.29611431 | 59.30 |
| ShWRKY96-1 | ShWRKY131-3 | 0.934783 | 0 | 0 | 0 | 0 | 0 | 0.00 |
| ShWRKY93-3 | ShWRKY107 | 1.094091 | 0 | 0 | 0 | 0 | 0 | 0.00 |
| ShWRKY74 | ShWRKY93-3 | 0.859464 | 1.164230385 | 0.738224951 | Purifying | 95.42872007 | 95.42872007 | 95.43 |
| ShWRKY132-2 | ShWRKY96-1 | 0.938753 | 0 | 0 | 0 | 0 | 0 | 0.00 |
| ShWRKY154-1 | ShWRKY6 | 0.636064 | 1.737713147 | 0.366035033 | Purifying | 142.4355039 | 142.4355039 | 142.44 |
| ShWRKY15-1 | ShWRKY38-2 | 0.211189 | 1.360501213 | 0.15522902 | Purifying | 111.5164929 | 111.5164929 | 111.52 |
| ShWRKY13-2 | ShWRKY27-3 | 0.958131 | 0 | 0 | 0 | 0 | 0 | 0.00 |
| ShWRKY52-1 | ShWRKY130 | 0.407892 | 0.841528163 | 0.484703648 | Purifying | 68.97771828 | 68.97771828 | 68.98 |

**Table S5** The detailed information about *ShWRKYs* orthologs.

| **Sr. no.** | **Query tem** | **String ID** | **Identity** | **Bitscore** | **Preferred name** | **Annotation** |
| --- | --- | --- | --- | --- | --- | --- |
| 1 | ShWRKY64-1 | 3702.AT1G68150.1 | 73.8 | 137.5 | WRKY9 | Probable WRKY transcription factor 9; Transcription factor. Interacts specifically with the W box (5'-(T)TGAC[CT]-3'), a frequently occurring elicitor- responsive cis-acting element (By similarity) |
| 2 | ShWRKY42-2 | 3702.AT2G38470.1 | 52.4 | 297.4 | WRKY33 | Probable WRKY transcription factor 33; Transcription factor. Interacts specifically with the W box (5'-TTGAC[CT]-3'), a frequently occurring elicitor-responsive cis-acting element. Involved in defense responses. Required for resistance to the necrotrophic fungal pathogen B. cinerea. Regulates the antagonistic relationship between defense pathways mediating responses to the bacterial pathogen P. syringae and the necrotrophic pathogen B. cinerea. Required for the phytoalexin camalexin synthesis following infection with B. cinerea. Acts as positive regulator of the camalexin biosynthetic gen [...] |
| 3 | ShWRKY154-2 | 3702.AT3G04670.1 | 38 | 217.6 | WRKY39 | Probable WRKY transcription factor 39; Transcription factor. Interacts specifically with the W box (5'-(T)TGAC[CT]-3'), a frequently occurring elicitor- responsive cis-acting element (By similarity) |
| 4 | ShWRKY124-2 | 3702.AT2G38470.1 | 61.1 | 282 | WRKY33 | Probable WRKY transcription factor 33; Transcription factor. Interacts specifically with the W box (5'-TTGAC[CT]-3'), a frequently occurring elicitor-responsive cis-acting element. Involved in defense responses. Required for resistance to the necrotrophic fungal pathogen *B. cinerea*. Regulates the antagonistic relationship between defense pathways mediating responses to the bacterial pathogen P. syringae and the necrotrophic pathogen *B. cinerea*. Required for the phytoalexin camalexin synthesis following infection with *B. cinerea*. Acts as positive regulator of the camalexin biosynthetic gen [...] |
| 5 | ShWRKY145-2 | 3702.AT4G31800.1 | 36.3 | 142.9 | WRKY18 | Pathogen-induced transcription factor. Binds W-box sequences in vitro. Forms protein complexes with itself and with WRKY40 and WRKY60. Constitutive expression of WRKY18 enhanced resistance to P. syringae, but its coexpression with WRKY40 or WRKY60 made plants more susceptible to both P. syringae and *B. cinerea*. WRKY18, WRKY40, and WRKY60 have partially redundant roles in response to the hemibiotrophic bacterial pathogen Pseudomonas syringae and the necrotrophic fungal pathogen Botrytis cinerea, with WRKY18 playing a more important role than the other two; Belongs to the WRKY group I [...] |
| 6 | ShWRKY143-3 | 3702.AT4G31800.1 | 38.5 | 144.8 | WRKY18 | Pathogen-induced transcription factor. Binds W-box sequences in vitro. Forms protein complexes with itself and with WRKY40 and WRKY60. Constitutive expression of WRKY18 enhanced resistance to P. syringae, but its coexpression with WRKY40 or WRKY60 made plants more susceptible to both *P. syringae* and *B. cinerea*. WRKY18, WRKY40, and WRKY60 have partially redundant roles in response to the hemibiotrophic bacterial pathogen *Pseudomonas syringae* and the necrotrophic fungal pathogen Botrytis cinerea, with WRKY18 playing a more important role than the other two; Belongs to the WRKY group I. |
| 7 | ShWRKY45-1 | 3702.AT3G58710.1 | 71 | 131.7 | WRKY69 | Probable WRKY transcription factor 69; Transcription factor. Interacts specifically with the W box (5'-(T)TGAC[CT]-3'), a frequently occurring elicitor- responsive cis-acting element (By similarity) |
| 8 | ShWRKY130 | 3702.AT1G62300.1 | 48.5 | 175.6 | WRKY6 | WRKY family transcription factor; Transcription factor involved in the control of processes related to senescence and pathogen defense. Interacts specifically with the W box (5'- (T)TGAC[CT]-3'), a frequently occurring elicitor-responsive cis- acting element. Activates the transcription of the SIRK gene and represses its own expression and that of the WRKY42 genes. Modulates phosphate homeostasis and Pi translocation by regulating PHO1 expression; Belongs to the WRKY group II-b family |
| 9 | ShWRKY115 | 3702.AT5G24110.1 | 41.9 | 66.2 | WRKY30 | Probable WRKY transcription factor 30; Transcription factor. Interacts specifically with the W box (5'-(T)TGAC[CT]-3'), a frequently occurring elicitor- responsive cis-acting element (By similarity) |
| 11 | ShWRKY54-1 | 3702.AT5G15130.1 | 44.9 | 197.2 | WRKY72 | Probable WRKY transcription factor 72; Transcription factor. Interacts specifically with the W box (5'-(T)TGAC[CT]-3'), a frequently occurring elicitor- responsive cis-acting element (By similarity) |
| 12 | ShWRKY69-2 | 3702.AT3G58710.1 | 57.4 | 119 | WRKY69 | Probable WRKY transcription factor 69; Transcription factor. Interacts specifically with the W box (5'-(T)TGAC[CT]-3'), a frequently occurring elicitor- responsive cis-acting element (By similarity) |
| 14 | ShWRKY82-2 | 3702.AT4G26440.1 | 43.6 | 301.2 | WRKY34 | Probable WRKY transcription factor 34; Transcription factor. Interacts specifically with the W box (5'-(T)TGAC[CT]-3'), a frequently occurring elicitor- responsive cis-acting element (By similarity) |
| 15 | ShWRKY19-2 | 3702.AT4G23810.1 | 46.8 | 109.4 | WRKY53 | Probable WRKY transcription factor 53; Transcription factor. Interacts specifically with the W box (5'-(T)TGAC[CT]-3'), a frequently occurring elicitor- responsive cis-acting element. May regulate the early events of leaf senescence. Negatively regulates the expression of ESR/ESP |
| 16 | ShWRKY19-3 | 3702.AT5G24110.1 | 41.9 | 113.2 | WRKY30 | Probable WRKY transcription factor 30; Transcription factor. Interacts specifically with the W box (5'-(T)TGAC[CT]-3'), a frequently occurring elicitor- responsive cis-acting element (By similarity) |
| 17 | ShWRKY58 | 3702.AT5G01900.1 | 40.5 | 65.1 | WRKY62 | Probable WRKY transcription factor 62; Transcription factor. Interacts specifically with the W box (5'-(T)TGAC[CT]-3'), a frequently occurring elicitor- responsive cis-acting element (By similarity); Belongs to the WRKY group III family |
| 18 | ShWRKY44-2 | 3702.AT5G22570.1 | 29.8 | 77.8 | WRKY38 | Probable WRKY transcription factor 38; Transcription factor. Interacts specifically with the W box (5'-(T)TGAC[CT]-3'), a frequently occurring elicitor- responsive cis-acting element (By similarity) |
| 19 | ShWRKY59-1 | 3702.AT5G22570.1 | 38.2 | 77 | WRKY38 | Probable WRKY transcription factor 38; Transcription factor. Interacts specifically with the W box (5'-(T)TGAC[CT]-3'), a frequently occurring elicitor- responsive cis-acting element (By similarity) |
| 20 | ShWRKY61-3 | 3702.AT1G29860.1 | 85.5 | 154.5 | WRKY71 | Probable WRKY transcription factor 71; Transcription factor. Interacts specifically with the W box (5'-(T)TGAC[CT]-3'), a frequently occurring elicitor- responsive cis-acting element (By similarity) |
| 21 | ShWRKY116-1 | 3702.AT4G18170.1 | 74 | 149.1 | WRKY28 | Probable WRKY transcription factor 28; Transcription factor. Interacts specifically with the W box (5'-(T)TGAC[CT]-3'), a frequently occurring elicitor- responsive cis-acting element (By similarity) |
| 22 | ShWRKY136-1 | 3702.AT5G52830.1 | 51.2 | 123.2 | WRKY27 | Encodes a WRKY transcription factor WRKY27. Mutation in Arabidopsis WRKY27 results in delayed symptom development in response to the bacterial wilt pathogen *Ralstonia solanacearum* |
| 23 | ShWRKY125-3 | 3702.AT2G38470.1 | 51.6 | 285 | WRKY33 | Probable WRKY transcription factor 33; Transcription factor. Interacts specifically with the W box (5'-TTGAC[CT]-3'), a frequently occurring elicitor-responsive cis-acting element. Involved in defense responses. Required for resistance to the necrotrophic fungal pathogen *B. cinerea*. Regulates the antagonistic relationship between defense pathways mediating responses to the bacterial pathogen *P. syringae* and the necrotrophic pathogen *B. cinerea*. Required for the phytoalexin camalexin synthesis following infection with *B. cinerea*. Acts as positive regulator of the camalexin biosynthetic gen [...] |
| 24 | ShWRKY124-1 | 3702.AT2G38470.1 | 54.7 | 283.5 | WRKY33 | Probable WRKY transcription factor 33; Transcription factor. Interacts specifically with the W box (5'-TTGAC[CT]-3'), a frequently occurring elicitor-responsive cis-acting element. Involved in defense responses. Required for resistance to the necrotrophic fungal pathogen *B. cinerea*. Regulates the antagonistic relationship between defense pathways mediating responses to the bacterial pathogen *P. syringae* and the necrotrophic pathogen *B. cinerea*. Required for the phytoalexin camalexin synthesis following infection with *B. cinerea*. Acts as positive regulator of the camalexin biosynthetic gen. |
| 25 | ShWRKY93-3 | 3702.AT4G31550.1 | 49.8 | 252.7 | WRKY11 | Probable WRKY transcription factor 11; Transcription factor. Interacts specifically with the W box (5'-(T)TGAC[CT]-3'), a frequently occurring elicitor- responsive cis-acting element (By similarity) |
| 26 | ShWRKY69-1 | 3702.AT3G58710.1 | 57.4 | 119 | WRKY69 | Probable WRKY transcription factor 69; Transcription factor. Interacts specifically with the W box (5'-(T)TGAC[CT]-3'), a frequently occurring elicitor- responsive cis-acting element (By similarity) |
| 27 | ShWRKY74 | 3702.AT2G34830.1 | 86 | 194.1 | WRKY35 | Probable WRKY transcription factor 35; Transcription factor. Interacts specifically with the W box (5'-(T)TGAC[CT]-3'), a frequently occurring elicitor- responsive cis-acting element (By similarity) |
| 28 | ShWRKY40-2 | 3702.AT1G13960.1 | 45.3 | 331.6 | WRKY4 | Probable WRKY transcription factor 4; Transcription factor that binds specifically to the W box (5'-(T)TGAC[CT]-3'), a frequently occurring elicitor- responsive cis-acting element. Has a positive role in resistance to necrotrophic pathogens (e.g. *Botrytis cinerea*), but a negative effect on plant resistance to biotrophic pathogens (e.g. *Pseudomonas syringae*) |
| 29 | ShWRKY96-1 | 3702.AT5G24110.1 | 37.2 | 72 | WRKY30 | Probable WRKY transcription factor 30; Transcription factor. Interacts specifically with the W box (5'-(T)TGAC[CT]-3'), a frequently occurring elicitor- responsive cis-acting element (By similarity) |
| 30 | ShWRKY68-3 | 3702.AT1G62300.1 | 44.9 | 246.1 | WRKY6 | WRKY family transcription factor; Transcription factor involved in the control of processes related to senescence and pathogen defense. Interacts specifically with the W box (5'- (T)TGAC[CT]-3'), a frequently occurring elicitor-responsive cis- acting element. Activates the transcription of the SIRK gene and represses its own expression and that of the WRKY42 genes. Modulates phosphate homeostasis and Pi translocation by regulating PHO1 expression; Belongs to the WRKY group II-b family |
| 31 | ShWRKY75-1 | 3702.AT5G52830.1 | 76 | 163.7 | WRKY27 | Encodes a WRKY transcription factor WRKY27. Mutation in Arabidopsis WRKY27 results in delayed symptom development in response to the bacterial wilt pathogen *Ralstonia solanacearum* |
| 32 | ShWRKY22-1 | 3702.AT5G24110.1 | 45.5 | 112.8 | WRKY30 | Probable WRKY transcription factor 30; Transcription factor. Interacts specifically with the W box (5'-(T)TGAC[CT]-3'), a frequently occurring elicitor- responsive cis-acting element (By similarity) |
| 33 | ShWRKY39-1 | 3702.AT4G23810.1 | 44.3 | 111.7 | WRKY53 | Probable WRKY transcription factor 53; Transcription factor. Interacts specifically with the W box (5'-(T)TGAC[CT]-3'), a frequently occurring elicitor- responsive cis-acting element. May regulate the early events of leaf senescence. Negatively regulates the expression of ESR/ESP |
| 34 | ShWRKY22-2 | 3702.AT2G46400.1 | 44.8 | 114.8 | WRKY46 | Probable WRKY transcription factor 46; Transcription factor involved in the regulation of osmotic stress responses and stomatal movement. Interacts specifically with the W box (5'-(T)TGAC[CT]-3'), a frequently occurring elicitor-responsive cis-acting element (By similarity) |
| 35 | ShWRKY127-1 | 3702.AT4G18170.1 | 86.4 | 153.7 | WRKY28 | Probable WRKY transcription factor 28; Transcription factor. Interacts specifically with the W box (5'-(T)TGAC[CT]-3'), a frequently occurring elicitor- responsive cis-acting element (By similarity) |
| 36 | ShWRKY154-1 | 3702.AT3G04670.1 | 38.4 | 220.7 | WRKY39 | Probable WRKY transcription factor 39; Transcription factor. Interacts specifically with the W box (5'-(T)TGAC[CT]-3'), a frequently occurring elicitor- responsive cis-acting element (By similarity) |
| 37 | ShWRKY38-2 | 3702.AT2G30590.1 | 43.9 | 259.2 | WRKY21 | Probable WRKY transcription factor 21; Transcription factor. Interacts specifically with the W box (5'-(T)TGAC[CT]-3'), a frequently occurring elicitor- responsive cis-acting element (By similarity) |
| 38 | ShWRKY29-1 | 3702.AT5G56270.1 | 34.8 | 187.6 | WRKY2 | Probable WRKY transcription factor 2; Transcription factor. Regulates WOX8 and WOX9 expression and basal cell division patterns during early embryogenesis. Interacts specifically with the W box (5'-(T)TGAC[CT]-3'), a frequently occurring elicitor-responsive cis-acting element. Required to repolarize the zygote from a transient symmetric state; Belongs to the WRKY group I family |
| 39 | ShWRKY15-1 | 3702.AT3G04670.1 | 48.7 | 263.8 | WRKY39 | Probable WRKY transcription factor 39; Transcription factor. Interacts specifically with the W box (5'-(T)TGAC[CT]-3'), a frequently occurring elicitor- responsive cis-acting element (By similarity) |
| 40 | ShWRKY42-3 | 3702.AT2G38470.1 | 52.7 | 297.7 | WRKY33 | Probable WRKY transcription factor 33; Transcription factor. Interacts specifically with the W box (5'-TTGAC[CT]-3'), a frequently occurring elicitor-responsive cis-acting element. Involved in defense responses. Required for resistance to the necrotrophic fungal pathogen *B. cinerea*. Regulates the antagonistic relationship between defense pathways mediating responses to the bacterial pathogen *P. syringae* and the necrotrophic pathogen *B. cinerea*. Required for the phytoalexin camalexin synthesis following infection with *B. cinerea*. Acts as positive regulator of the camalexin biosynthetic gen [...] |
| 41 | ShWRKY81-1 | 3702.AT2G44745.1 | 81.4 | 171.8 | WRKY12 | Probable WRKY transcription factor 12; Transcription factor. Interacts specifically with the W box (5'-(T)TGAC[CT]-3'), a frequently occurring elicitor- responsive cis-acting element (By similarity) |
| 42 | ShWRKY43 | 3702.AT2G38470.1 | 51 | 295 | WRKY33 | Probable WRKY transcription factor 33; Transcription factor. Interacts specifically with the W box (5'-TTGAC[CT]-3'), a frequently occurring elicitor-responsive cis-acting element. Involved in defense responses. Required for resistance to the necrotrophic fungal pathogen *B. cinerea*. Regulates the antagonistic relationship between defense pathways mediating responses to the bacterial pathogen P. syringae and the necrotrophic pathogen B.cinerea. Required for the phytoalexin camalexin synthesis following infection with *B. cinerea*. Acts as positive regulator of the camalexin biosynthetic gen [...] |
| 43 | ShWRKY131-3 | 3702.AT5G22570.1 | 46.4 | 65.9 | WRKY38 | Probable WRKY transcription factor 38; Transcription factor. Interacts specifically with the W box (5'-(T)TGAC[CT]-3'), a frequently occurring elicitor- responsive cis-acting element (By similarity) |
| 44 | ShWRKY52-1 | 3702.AT4G01720.1 | 44.5 | 144.4 | WRKY47 | Probable WRKY transcription factor 47; Transcription factor. Interacts specifically with the W box (5'-(T)TGAC[CT]-3'), a frequently occurring elicitor- responsive cis-acting element (By similarity) |
| 45 | ShWRKY72-2 | 3702.AT1G29860.1 | 51.8 | 153.3 | WRKY71 | Probable WRKY transcription factor 71; Transcription factor. Interacts specifically with the W box (5'-(T)TGAC[CT]-3'), a frequently occurring elicitor- responsive cis-acting element (By similarity) |
| 46 | ShWRKY2-1 | 3702.AT1G13960.1 | 42.7 | 303.5 | WRKY4 | Probable WRKY transcription factor 4; Transcription factor that binds specifically to the W box (5'-(T)TGAC[CT]-3'), a frequently occurring elicitor- responsive cis-acting element. Has a positive role in resistance to necrotrophic pathogens (e.g. *Botrytis cinerea*), but a negative effect on plant resistance to biotrophic pathogens (e.g. *Pseudomonas syringae*) |
| 47 | ShWRKY146-1 | 3702.AT4G23810.1 | 54.1 | 126.3 | WRKY53 | Probable WRKY transcription factor 53; Transcription factor. Interacts specifically with the W box (5'-(T)TGAC[CT]-3'), a frequently occurring elicitor- responsive cis-acting element. May regulate the early events of leaf senescence. Negatively regulates the expression of ESR/ESP |
| 48 | ShWRKY77-2 | 3702.AT1G68150.1 | 46.7 | 172.2 | WRKY9 | Probable WRKY transcription factor 9; Transcription factor. Interacts specifically with the W box (5'-(T)TGAC[CT]-3'), a frequently occurring elicitor- responsive cis-acting element (By similarity) |
| 49 | ShWRKY29-2 | 3702.AT5G56270.1 | 35.2 | 185.3 | WRKY2 | Probable WRKY transcription factor 2; Transcription factor. Regulates WOX8 and WOX9 expression and basal cell division patterns during early embryogenesis. Interacts specifically with the W box (5'-(T)TGAC[CT]-3'), a frequently occurring elicitor-responsive cis-acting element. Required to repolarize the zygote from a transient symmetric state; Belongs to the WRKY group I family |
| 50 | ShWRKY126 | 3702.AT5G43290.1 | 56.8 | 134 | WRKY49 | Probable WRKY transcription factor 49; Transcription factor. Interacts specifically with the W box (5'-(T)TGAC[CT]-3'), a frequently occurring elicitor- responsive cis-acting element (By similarity) |
| 51 | ShWRKY68-4 | 3702.AT1G62300.1 | 50.5 | 231.1 | WRKY6 | WRKY family transcription factor; Transcription factor involved in the control of processes related to senescence and pathogen defense. Interacts specifically with the W box (5'- (T)TGAC[CT]-3'), a frequently occurring elicitor-responsive cis- acting element. Activates the transcription of the SIRK gene and represses its own expression and that of the WRKY42 genes. Modulates phosphate homeostasis and Pi translocation by regulating PHO1 expression; Belongs to the WRKY group II-b family |
| 52 | ShWRKY3-2 | 3702.AT2G34830.1 | 89.2 | 150.2 | WRKY35 | Probable WRKY transcription factor 35; Transcription factor. Interacts specifically with the W box (5'-(T)TGAC[CT]-3'), a frequently occurring elicitor- responsive cis-acting element (By similarity) |
| 53 | ShWRKY49-3 | 3702.AT4G23810.1 | 48.8 | 133.7 | WRKY53 | Probable WRKY transcription factor 53; Transcription factor. Interacts specifically with the W box (5'-(T)TGAC[CT]-3'), a frequently occurring elicitor- responsive cis-acting element. May regulate the early events of leaf senescence. Negatively regulates the expression of ESR/ESP |
| 54 | ShWRKY147 | 3702.AT5G15130.1 | 36.7 | 202.6 | WRKY72 | Probable WRKY transcription factor 72; Transcription factor. Interacts specifically with the W box (5'-(T)TGAC[CT]-3'), a frequently occurring elicitor- responsive cis-acting element (By similarity) |
| 55 | ShWRKY138 | 3702.AT1G62300.1 | 71 | 145.6 | WRKY6 | WRKY family transcription factor; Transcription factor involved in the control of processes related to senescence and pathogen defense. Interacts specifically with the W box (5'- (T)TGAC[CT]-3'), a frequently occurring elicitor-responsive cis- acting element. Activates the transcription of the SIRK gene and represses its own expression and that of the WRKY42 genes. Modulates phosphate homeostasis and Pi translocation by regulating PHO1 expression; Belongs to the WRKY group II-b family |
| 56 | ShWRKY13-2 | 3702.AT2G46400.1 | 44.3 | 84.3 | WRKY46 | Probable WRKY transcription factor 46; Transcription factor involved in the regulation of osmotic stress responses and stomatal movement. Interacts specifically with the W box (5'-(T)TGAC[CT]-3'), a frequently occurring elicitor-responsive cis-acting element (By similarity) |
| 57 | ShWRKY55 | 3702.AT5G43290.1 | 56.8 | 134.4 | WRKY49 | Probable WRKY transcription factor 49; Transcription factor. Interacts specifically with the W box (5'-(T)TGAC[CT]-3'), a frequently occurring elicitor- responsive cis-acting element (By similarity) |
| 58 | ShWRKY27-3 | 3702.AT4G26640.2 | 45.9 | 286.2 | WRKY20 | WRKY family transcription factor family protein; Transcription factor. Interacts specifically with the W box (5'-(T)TGAC[CT]-3'), a frequently occurring elicitor- responsive cis-acting element (By similarity) |
| 59 | ShWRKY15-3 | 3702.AT3G04670.1 | 50.8 | 219.5 | WRKY39 | Probable WRKY transcription factor 39; Transcription factor. Interacts specifically with the W box (5'-(T)TGAC[CT]-3'), a frequently occurring elicitor- responsive cis-acting element (By similarity) |
| 60 | ShWRKY105 | 3702.AT1G13960.1 | 43.5 | 216.1 | WRKY4 | Probable WRKY transcription factor 4; Transcription factor that binds specifically to the W box (5'-(T)TGAC[CT]-3'), a frequently occurring elicitor- responsive cis-acting element. Has a positive role in resistance to necrotrophic pathogens (e.g. *Botrytis cinerea*), but a negative effect on plant resistance to biotrophic pathogens (e.g. *Pseudomonas syringae*) |
| 61 | ShWRKY44-1 | 3702.AT5G22570.1 | 29.8 | 77.8 | WRKY38 | Probable WRKY transcription factor 38; Transcription factor. Interacts specifically with the W box (5'-(T)TGAC[CT]-3'), a frequently occurring elicitor- responsive cis-acting element (By similarity) |
| 62 | ShWRKY6 | 3702.AT4G24240.1 | 49.1 | 144.4 | WRKY7 | Probable WRKY transcription factor 7; Transcription factor. Interacts specifically with the W box (5'-(T)TGAC[CT]-3'), a frequently occurring elicitor- responsive cis-acting element (By similarity); Belongs to the WRKY group II-d family |
| 63 | ShWRKY77-3 | 3702.AT1G68150.1 | 45.4 | 171.8 | WRKY9 | Probable WRKY transcription factor 9; Transcription factor. Interacts specifically with the W box (5'-(T)TGAC[CT]-3'), a frequently occurring elicitor- responsive cis-acting element (By similarity) |
| 64 | ShWRKY75-3 | 3702.AT5G52830.1 | 76 | 164.1 | WRKY27 | Encodes a WRKY transcription factor WRKY27. Mutation in Arabidopsis WRKY27 results in delayed symptom development in response to the bacterial wilt pathogen *Ralstonia solanacearum* |
| 65 | ShWRKY118-1 | 3702.AT2G46400.1 | 51.5 | 89 | WRKY46 | Probable WRKY transcription factor 46; Transcription factor involved in the regulation of osmotic stress responses and stomatal movement. Interacts specifically with the W box (5'-(T)TGAC[CT]-3'), a frequently occurring elicitor-responsive cis-acting element (By similarity) |
| 66 | ShWRKY118-2 | 3702.AT2G46400.1 | 53 | 90.5 | WRKY46 | Probable WRKY transcription factor 46; Transcription factor involved in the regulation of osmotic stress responses and stomatal movement. Interacts specifically with the W box (5'-(T)TGAC[CT]-3'), a frequently occurring elicitor-responsive cis-acting element (By similarity) |


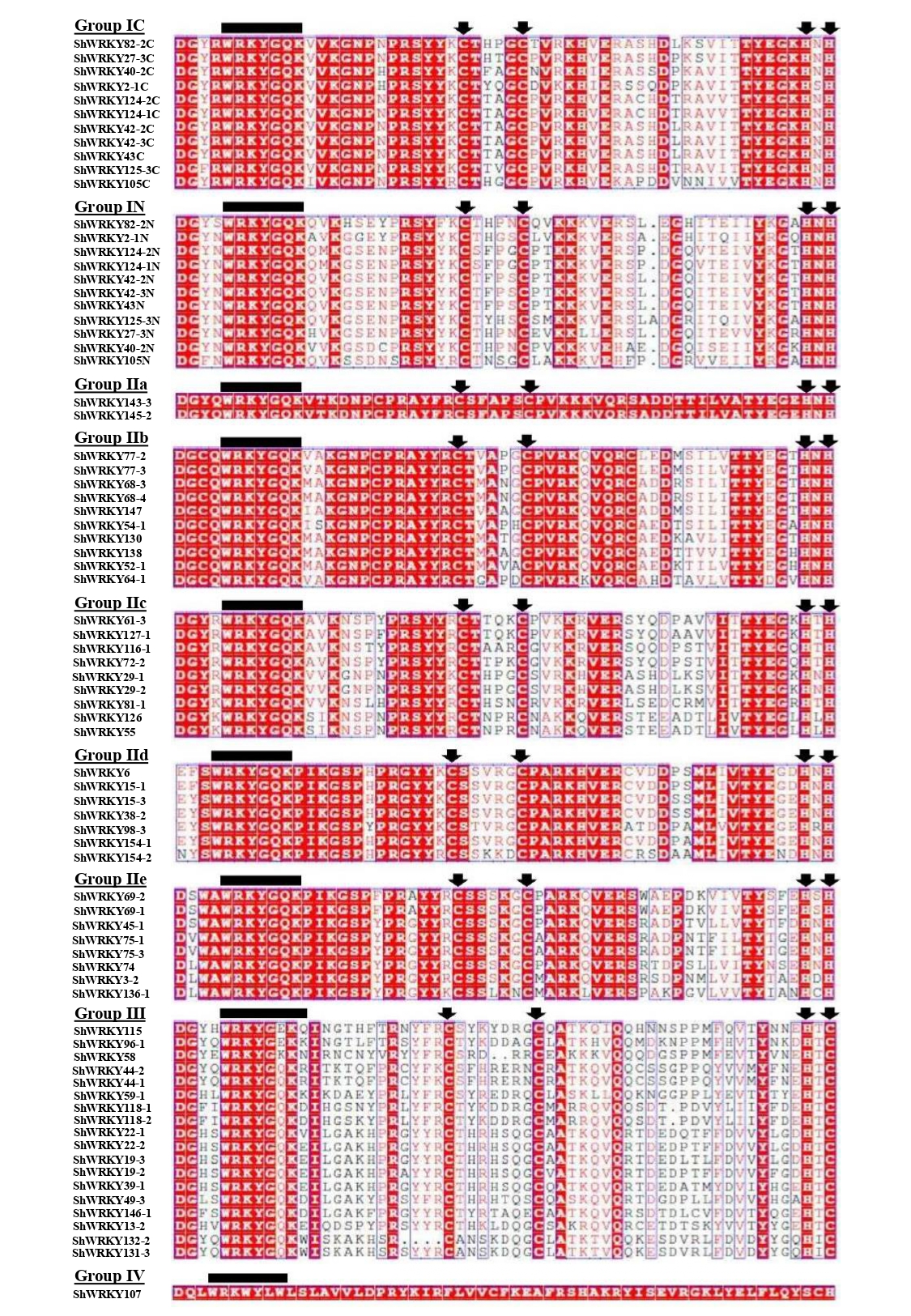


**Figure S1** Alignment of WRKYs domain amino acid sequences of *Saccharum* spp. hybrid R570. ‘C’ and ‘N’ indicate the C-terminal and N-terminal WRKY domains, respectively.


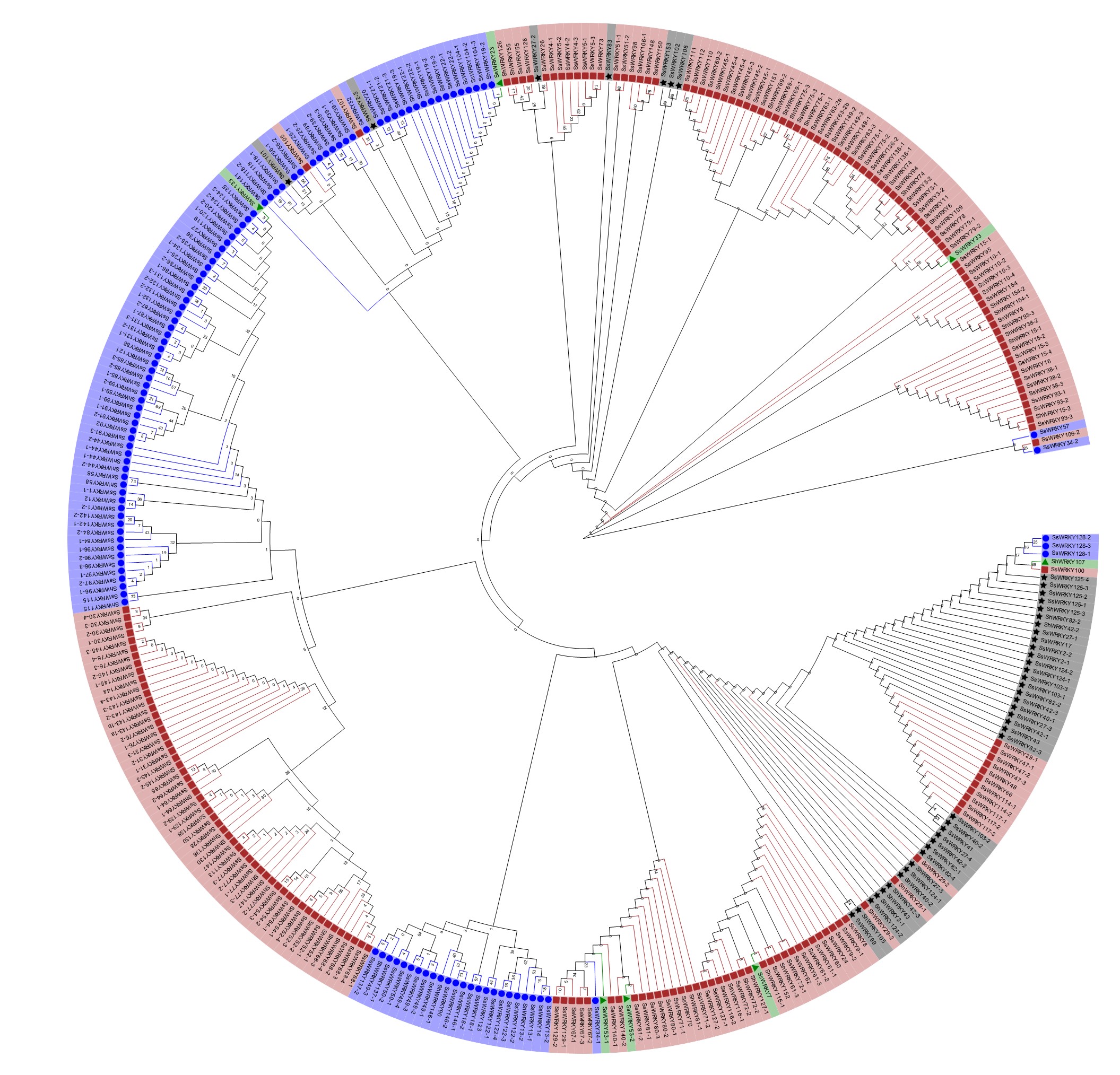


**Figure S2** Phylogenetic tree of WRKY domains from *Saccharum* spp. hybrid R570 and *S. spontaneum* (AP85-441). The unrooted NJ tree was constructed based on the WRKY domains from *Saccharum* spp. hybrid R570 and *S. spontaneum* (AP85-441) using MEGA7.0 with bootstraps of 1000 replicates.


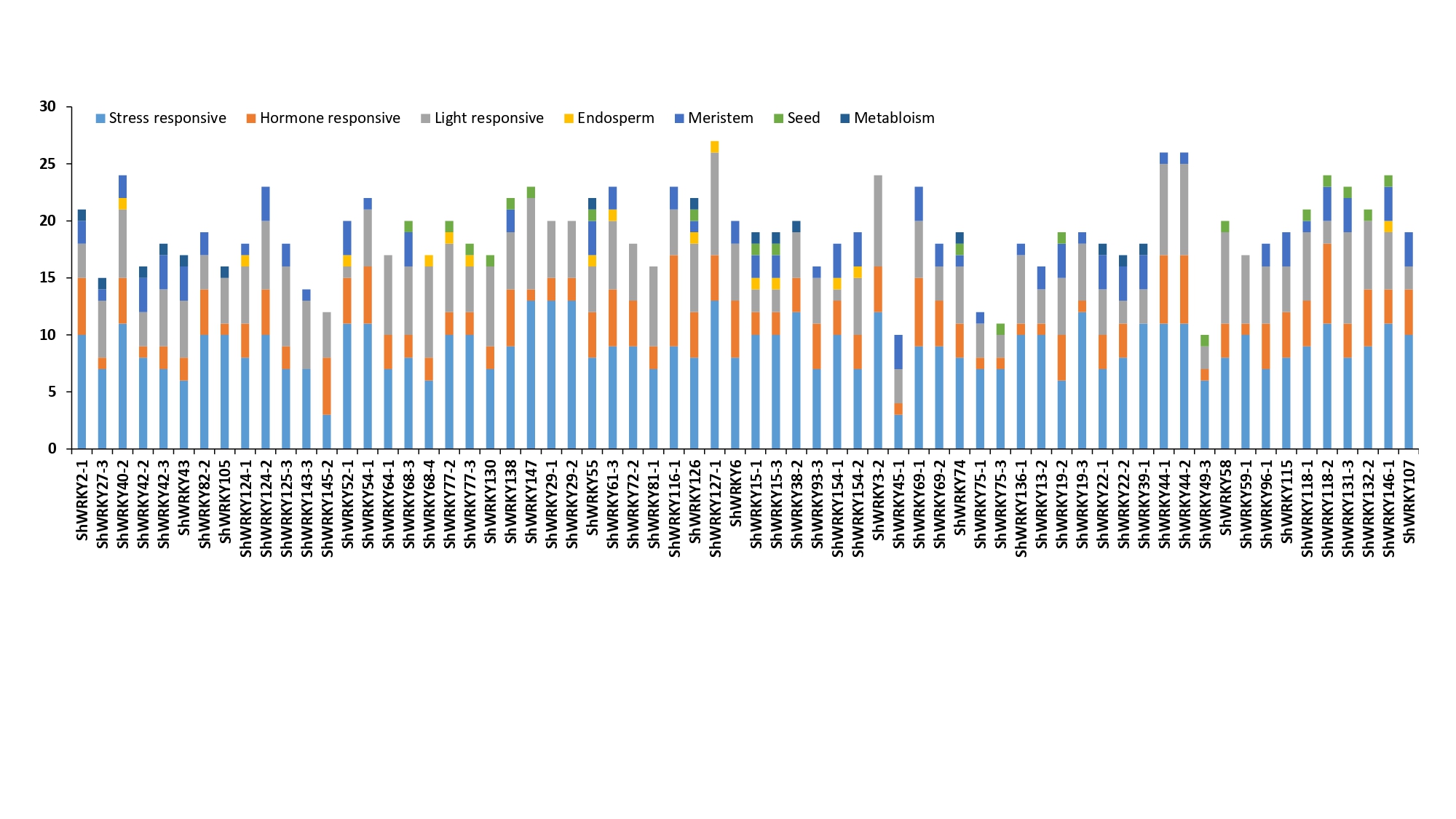


**Figure S3** The *cis*-elements in the promoter of *Saccharum* spp. hybrid R570 WRKY gene family. The number of *cis*-elements related to stress, hormone, light, endosperm, meristem, seed and metabolism are presented in bars.


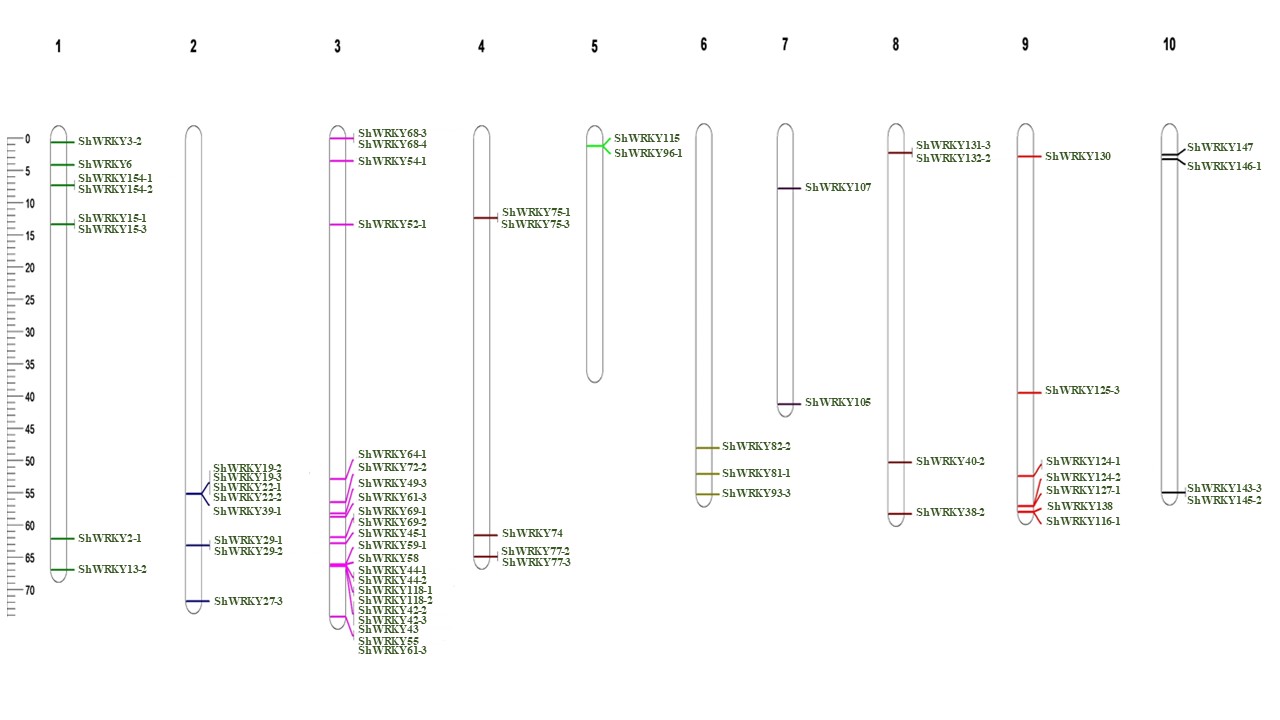


**Figure S4** Distribution of *WRKYs* on *Saccharum* spp. hybrid R570 chromosomes. In total, 66 *ShWRKYs* were mapped to 10 chromosomes. The scale (Mb) indicates the size of various chromosomes.


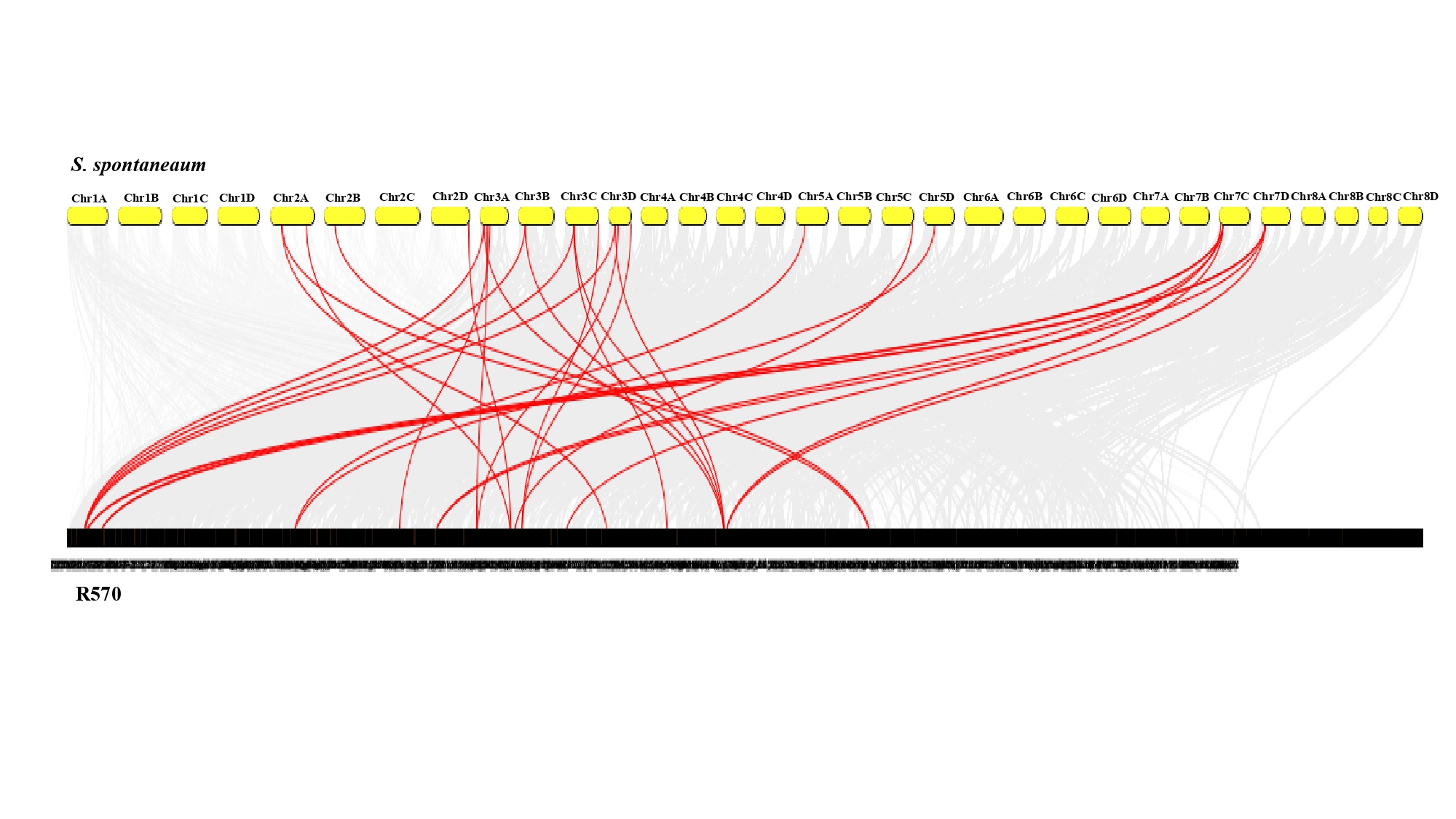


**Figure S5** Collinearity analysis among *Saccharum* spp. hybrid R570 and *S. spontaneaum*. Chromosomes of R570 and *S. spontaneaum* are indicated by yellow and brown boxes, respectively. Red lines indicates collinearity.


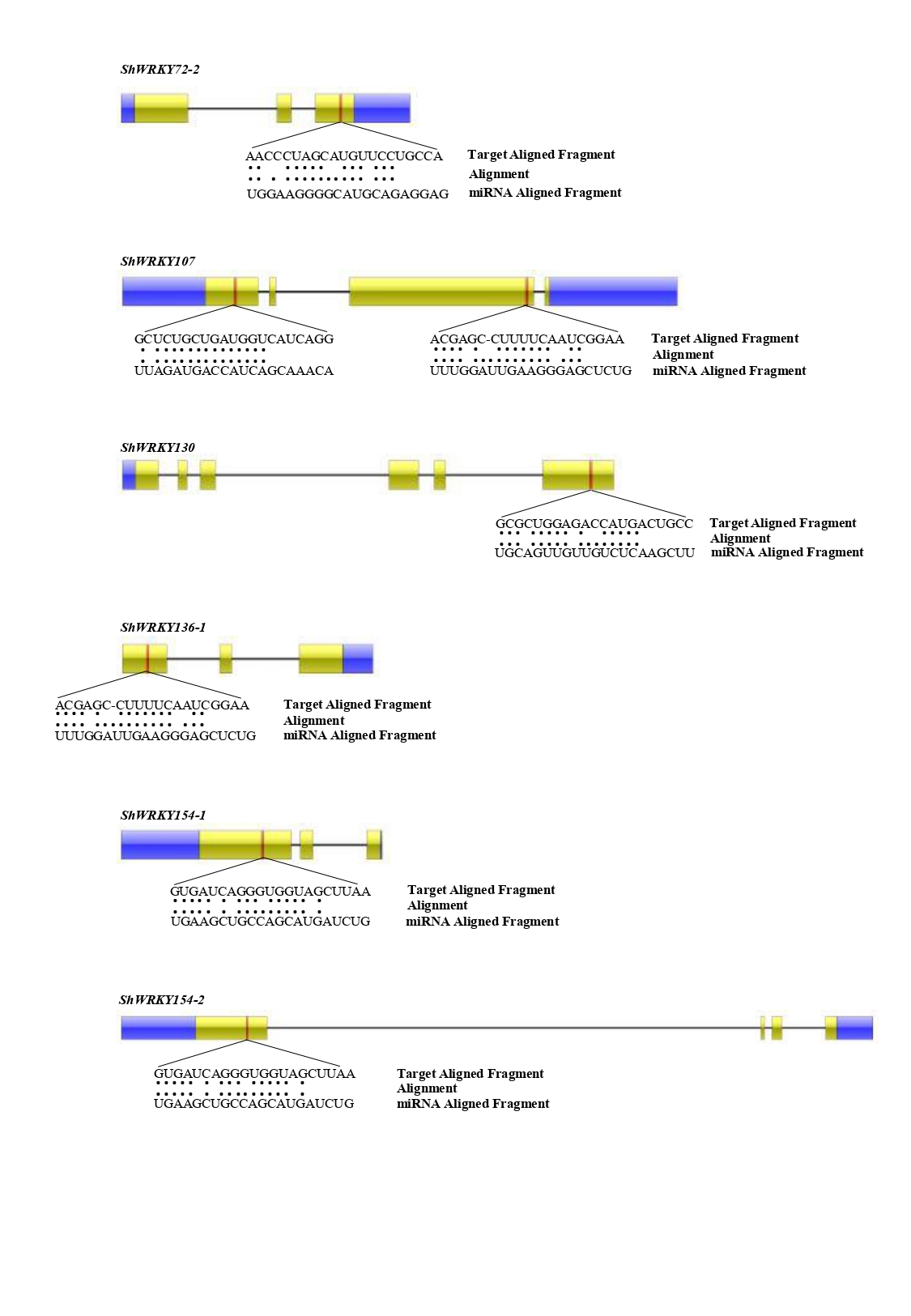


**Figure S6** Prediction of miRNA target site with target aligned fragment. Yellow and blue boxes represents exons and UTRs, respectively. Introns are given by black lines.


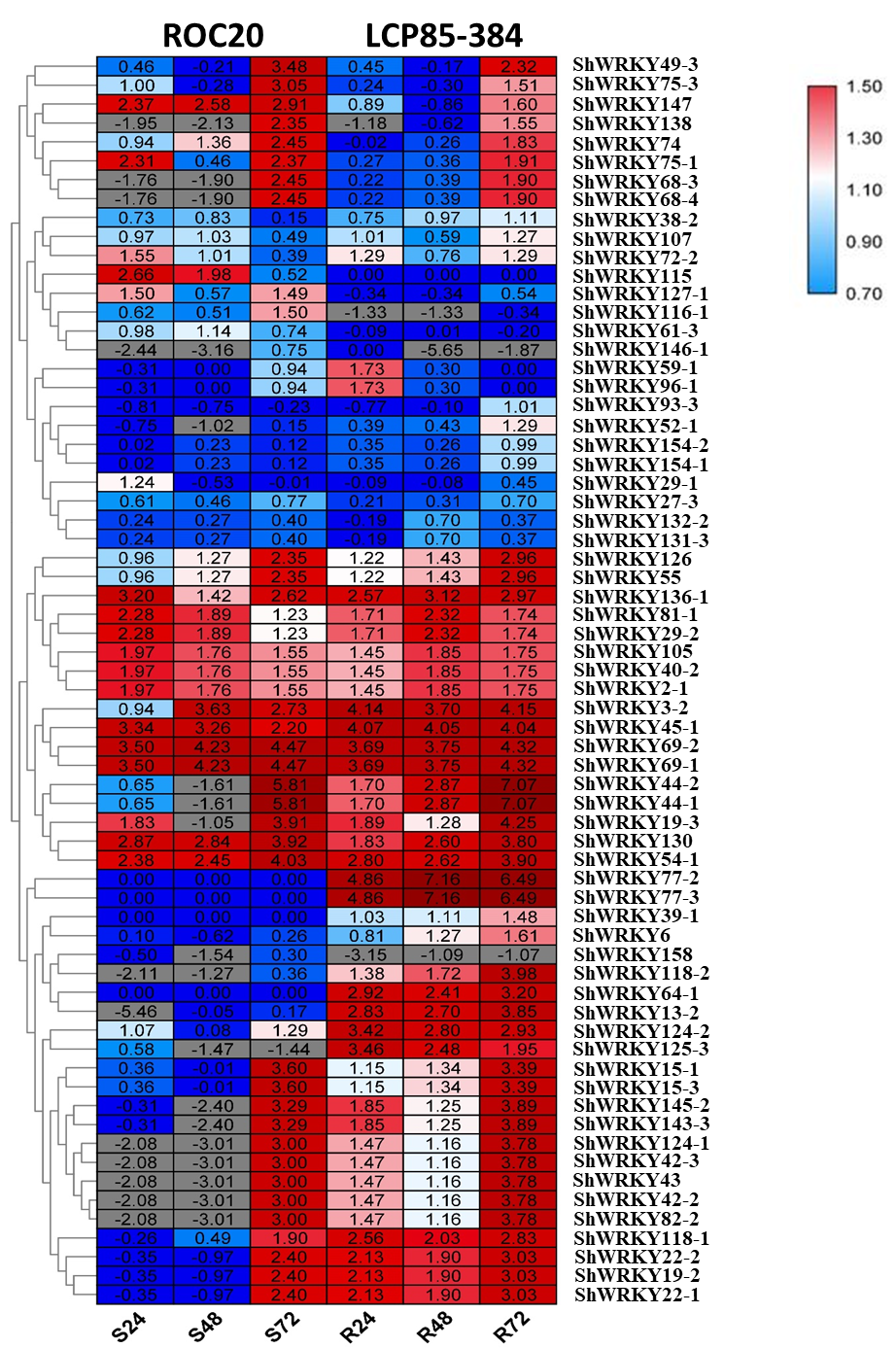


**Figure S7** Heat map of *Saccharum* spp. hybrid R570 WRKYs expression in transcriptome dataset of susceptible (ROC20) and resistant (LCP85-384) sugarcane cultivars against *Xanthomonas albilineans* infection.
